# Mixed Inhibition of cPEPCK by Genistein, Using an Extended Binding Site Located Adjacent to Its Catalytic Cleft

**DOI:** 10.1371/journal.pone.0141987

**Published:** 2015-11-03

**Authors:** Shashank Prakash Katiyar, Arpit Jain, Jaspreet Kaur Dhanjal, Durai Sundar

**Affiliations:** Department of Biochemical Engineering and Biotechnology, Indian Institute of Technology (IIT) Delhi, Hauz Khas, New Delhi, India; Bioinformatics Institute, SINGAPORE

## Abstract

Cytosolic phosphoenolpyruvate carboxykinase (cPEPCK) is a critical enzyme involved in gluconeogenesis, glyceroneogenesis and cataplerosis. cPEPCK converts oxaloacetic acid (OAA) into phosphoenol pyruvate (PEP) in the presence of GTP. cPEPCK is known to be associated with type 2 diabetes. Genistein is an isoflavone compound that shows anti-diabetic and anti-obesitic properties. Experimental studies have shown a decrease in the blood glucose level in the presence of genistein by lowering the functional activity of cPEPCK, an enzyme of gluconeogenesis. Using computational techniques such as molecular modeling, molecular docking, molecular dynamics simulation and binding free energy calculations, we identified cPEPCK as a direct target of genistein. We studied the molecular interactions of genistein with three possible conformations of cPEPCK—unbound cPEPCK (u_cPEPCK), GTP bound cPEPCK (GTP_cPEPCK) and GDP bound cPEPCK (GDP_cPEPCK). Binding of genistein was also compared with an already known cPEPCK inhibitor. We analyzed the interactions of genistein with cPEPCK enzyme and compared them with its natural substrate (OAA), product (PEP) and known inhibitor (3-MPA). Our results demonstrate that genistein uses the mechanism of mixed inhibition to block the functional activity of cPEPCK and thus can serve as a potential anti-diabetic and anti-obesity drug candidate. We also identified an extended binding site in the catalytic cleft of cPEPCK which is used by 3-MPA to inhibit cPEPCK non-competitively. We demonstrate that extended binding site of cPEPCK can further be exploited for designing new drugs against cPEPCK.

## Introduction

Phosphoenolpyruvate carboxykinase (PEPCK) enzyme catalyzes the reversible decarboxylation of oxaloacetic acid (OAA) to phosphoenolpyruvate (PEP) in the presence of guanosine triphosphate (GTP). In humans, two isoforms of PEPCK are expressed, namely cytosolic PEPCK (cPEPCK) and mitochondrial PEPCK (mPEPCK). cPEPCK is associated with gluconeogenesis [1, 2], glyceroneogenesis [3–5] and cataplerosis [6, 7]. It has been reported in previous studies that knocking out cPEPCK gene in mouse significantly decreases the level of glycogen and glucose in the host, leading to hypoglycemia and subsequent death [8, 9]. Also the over-expression of this enzyme results in type 2 diabetes with phenotypes such as obesity and fatty acid re-esterification [10, 11]. Type 2 diabetic and obese patients do not respond to insulin treatment and silencing of pck1 gene (PEPCK1 gene) increases the sensitivity to insulin in diabetic patients. Therefore, cPEPCK has been suggested as a potential therapeutic target for type 2 diabetes [12]. Despite being gluconeogenic in liver, many biochemical and metabolic tracer studies have shown cPEPCK to be glyceroneogenic too (mostly in adipose tissue) [13–15]. Over-expression of cPEPCK in adipose tissue has been reported to increase the fatty acid re-esterification, leading to obesity [16]. Hence, these studies prove cPEPCK to be a promising target against obesity along with type 2 diabetes.

The conversion of substrate OAA into product PEP by cPEPCK is accomplished in the presence of GTP, which transfers its γ-phosphate and gets converted to GDP. The structure of cPEPCK has been solved experimentally, captured in various active site conformations, such as holo-enzyme, GTP bound conformation, and product bound conformation [17]. Availability of the molecular structure of cPEPCK provides an excellent opportunity to study its molecular interactions with its substrate, product and inhibitor molecules. *In-silico* molecular docking, molecular dynamics simulations and binding free energy calculations can be used to investigate the interaction of a protein with its ligand [18].

A naturally occurring isoflavone compound called genistein, obtained from root peel extract of lupin, fava beans, soybeans, kudzu, medicinal plant *Flemingia vestita* and *Flemingia macrophylla*, has been shown to exhibit efficient antioxidant [19–22], anthelminthic [23–30] and anti-cancerous properties [31–36]. It has also been reported to show strong anti-diabetic and anti-obesity properties [37–41]. Furthermore, it was observed that the activity of cPEPCK was higher in the control group as compared to the genistein treated group, which resulted in decrease in blood glucose level in the treatment group, confirming genistein’s efficacy against type 2 diabetes [39, 40]. Lipid synthesis level was also found to be hindered in subjects treated with genistein [39, 40]. Hence, genistein is believed to interact with cPEPCK to suppress its activity. Anti-diabetic and anti-obesity properties of genistein are attributed to its ability to interact and inhibit the functional activity of cPEPCK [39, 40]. Though previous reports have indicated the role of genistein in suppressing the activity of cPEPCK, the molecular mode of its inhibition by genistein has not been studied yet.

In this study, we have reported the molecular interactions of genistein with cPEPCK enzyme using various well established computational methods to identify the binding mode and binding energy between the two molecules. Naturally, cPEPCK may exist in free, GDP bound and GTP bound states, and presence or absence of cofactor at the binding site may significantly affect the binding of substrate or inhibitor at the binding site. Hence, we used cPEPCK in all the three different binding conformational states to study their structural stability, binding of genistein at their active site and preference of each for genistein over their natural substrate. The objective of our work was thus to study the mechanism of cPEPCK functional activity inhibition by genistein by investigating its behavior within three different possible binding site environments of the human cPEPCK enzyme. We have also studied the action mechanism of 3-MPA, a known inhibitor of cPEPCK and compared it with the mechanism of genistein.

## Computational Methods

### cPEPCK structure refinement and binding site identification

The crystal structure for human cPEPCK was obtained from Protein Data Bank (PDB ID: 1KHG). The structure obtained was incomplete with few missing residues. Therefore, the protein structure was completed and refined using MODELLER 9.1 [42]. Binding site of cPEPCK was identified based on its crystallized substrate/cofactor bound conformations. The PDB structure 1KHE was used to identify GTP binding site and PDB structure 1KHF was used to identify the substrate and product binding sites. Volume and shape analysis of the binding sites was done using SiteMap, Version 3.3, package of Schrödinger 2014 [43].

### Different binding conformations of cPEPCK protein

The first step was to superimpose various binding site conformations of cPEPCK enzyme (PDB IDs: 1KHG, 1KHF and 1KHE) on each other to identify the differences in the three dimensional position of residues lining the active site. Later, the three conformations of cPEPCK; cPEPCK without cofactors (u_cPEPCK), cPEPCK bound with GTP (GTP_cPEPCK) and cPEPCK bound with GDP (GDP_cPEPCK) were generated by superimposing the generated cPEPCK structure (no missing residues) with PDB structures co-crystallized with GTP inhibitor and GDP (PDB ID: 1KHB and 1KHE). The structures obtained were then minimized to remove steric clashes using OPLS2005 force field in Maestro interface, version 9.6 and Desmond suite, version 3.6 [44–48].

### Molecular Docking of the three cPEPCK conformations with Genistein

**Fig 1:**
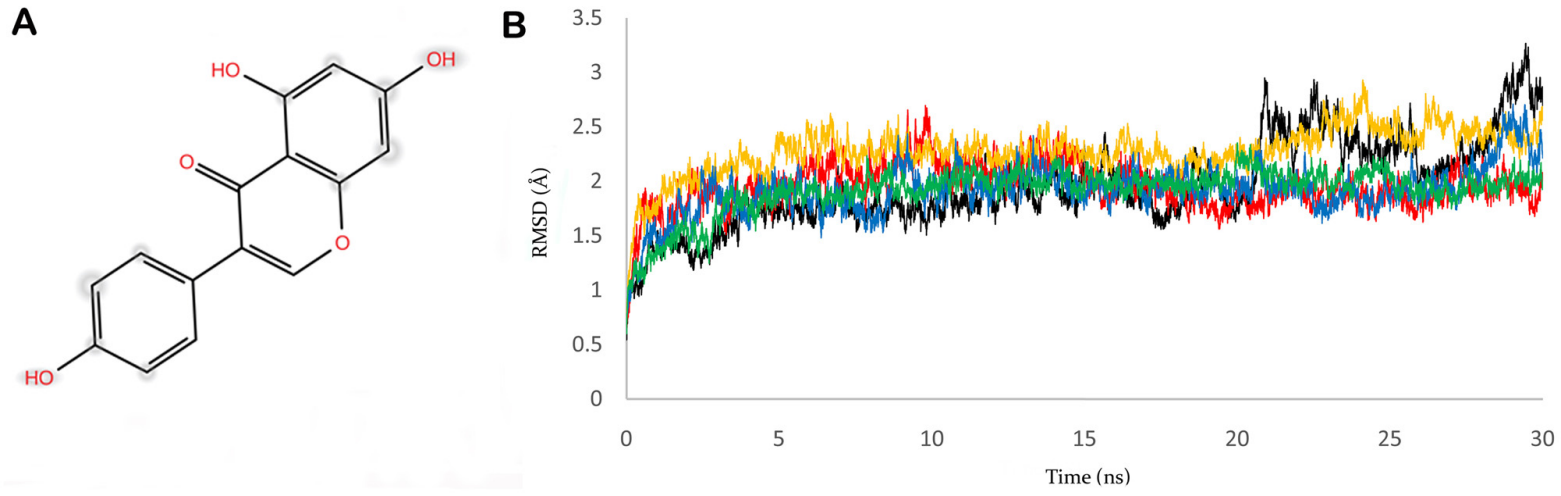
Structure of genistein and stability of cPEPCK protein. A) 2D representation of genistein's structure. B) RMSD graph of refined cPEPCK protein backbone (black), backbone of u_cPEPCK-genistein complex (red), backbone of GTP_cPEPCK-genistein complex (blue), backbone of GDP_cPEPCK-genistein complex (yellow), and backbone of OAA-GTP_cPEPCK protein-ligand complex (green).

AutoDock 4.2 suite was used to perform protein-ligand semi-flexible docking [49]. Molecular structure of genistein was retrieved from ligand database of PDB (Fig 1A). The ligands were prepared using MGLTools by defining the number of torsions and conversion into pdbqt format [50]. Similarly, MGLTools was used to prepare u_cPEPCK, GDP_cPEPCK, and GTP_cPEPCK by assigning AutoDock4 atom types, adding Gasteiger charges and conversion into pdbqt file format. Grid files were generated to define the binding locations of the ligands within all three conformations of cPEPCK protein using autogrid program of Autodock 4.2. In u_cPEPCK and GTP_cPEPCK, genistein was set to bind at substrate-binding site, while for GDP_cPEPCK, genistein was docked within the product-binding site. Once grid files were generated, autodock program generated several docked conformations of the ligand at its corresponding binding site on cPEPCK protein using Lamarckian Genetic Algorithm and the Empirical Binding Free Energy Function [51]. Default parameters of autodock were used for performing docking experiments. Similarly, cPEPCK’s natural substrate OAA and natural product PEP were also docked to GTP_cPEPCK and GDP_cPEPCK. OAA was docked with human cPEPCK to form a complex because of the absence of experimentally solved structure while PEP was rescored with cPEPCK using an experimental structure (PDB ID: 1KHF). OAA was docked with human cPEPCK using the binding site of OAA in rat cPEPCK (PDB ID: 2QF2) obtained by superimposition of human cPEPCK and rat cPEPCK structure. PEP was just re-scored using AutoDock at its own binding site in 1KHF and GDP was superimposed in cPEPCK cavity during rescoring. Re-scoring of PEP at its own binding site in 1KHF gave a docking score for the experimentally solved pose of PEP. Molecular structures of OAA and PEP were also obtained from the ligand database of PDB.

### Ligand binding refinement using PELE web server

PELE algorithm is based on steered stochastic approach along with protein structure prediction methods and is capable of projecting ligand migration dynamics within the protein [52, 53]. Docked conformation of genistein in u_cPEPCK state was further refined using ‘Ligand binding refinement’ protocol to include induced fit effect. The refinement allowed genistein to bind at its most preferred location within the binding cavity of u_cPEPCK.

### Molecular Dynamics Simulation

GPU accelerated Amber molecular dynamics suite with Amber ff99SB protein force field was used to perform all atoms explicit molecular dynamics simulations (MD simulations). Four 30 ns molecular dynamics simulations were performed: (i) to stabilize the refined structure of modeled cPEPCK protein; (ii) to account for the stability of genistein docked to u_cPEPCK; (iii) to study the stability and movements of genistein within the binding site of GTP_cPEPCK; and (iv) to investigate the dynamics of genistein and its interactions with GDP_cPEPCK. Also, two 45ns and 50ns MD simulations were carried out to study the dynamics of cPEPCK with its known inhibitor and genistein at inhibitor binding site. All proteins and protein-ligand complexes for MD simulations were prepared by adding hydrogens using Tleap module in AMBER 12 software package [51]. Antechamber program was used to generate force field parameters for the ligand as described by the general AMBER force fields (GAFF) [54]. The FF99SB force field was used to define parameters for cPEPCK protein and its complex with ligand [55]. All prepared proteins and protein-ligand complexes were solvated using TIP3P water model [56] in an octahedron box at 10Å distance around the protein. It was then followed by neutralization of systems by adding appropriate number of counter ions. The prepared neutral systems were further minimized, heated and equilibrated before running production MD simulations.

All prepared systems were minimized in two steps; firstly, the water molecules were minimized keeping force restraints over protein and complexes, which was then followed by minimization of whole system in the second step. The first 3000 steps of energy minimization were run with steepest descent method and the remaining 2000 steps with conjugate gradient method. Particle Mesh Ewald (PME) summation was used to handle the long-range Coulombic interactions with cutoff value of 10Å [57]. Minimized systems were then slowly heated to bring system’s temperature from 0K to 300K in NVT ensemble with time step of 0.005fs. Systems were then equilibrated until pressure and density of systems were stabilized in NPT ensemble. For equilibration and subsequent steps, Berendsen thermostat was used in the isothermal isobaric (NPT) ensemble with target pressure of 1 bar and pressure coupling constant of 2ps. Further, the production phase of the MD was run using same conditions for 30 ns. The SHAKE algorithm [58] was turned on for all atoms covalently bonded to a hydrogen atom which allowed an integrative time step of 2fs. Minimization, heating and equilibration of genistein-cPEPCK complexes were run using a small force restraint of 10 kcal/mol/Å^2^ over genistein.

During the MD simulations, root mean square deviation (RMSD), change in number of hydrogen bonds between ligand and protein, along with some other statistical analysis were calculated using VMD software [59]. RMSD of protein and the docked ligand within the binding pocket of protein were calculated for the entire simulation trajectory with reference to the initial structure. Images and interaction diagrams were prepared with free maestro molecular modeling environment [47]. Interactions between protein and ligands were analyzed by LIGPLOT v.4.0 using the default criteria of hydrogen bond identification [60].

### Binding free energy calculations using AMBER MMGB/PBSA

MMGB/PBSA calculations were performed using AMBER 12 software package over protein-ligand complex structures of stable trajectory obtained after MD simulation [61, 62]. Multiple structures of the protein-ligand complexes were generated by extracting 10ps spaced snapshots from stable trajectory of MD production run. Ions and water molecules were removed from the extracted structures. The free energy was individually calculated for protein, ligand and protein-ligand complexes. Finally, the binding energy was computed using the following formula:

$$\Delta {\text{G}}_{bind}=\;{\text{G}}_{complex}-\;{\text{G}}_{protein}-\;\Delta {\text{G}}_{ligand}$$

Where, G_complex_, G_protein_ and G_ligand_ are the calculated average free energies for each species solved using MMGB/PBSA method:

$$\Delta {\text{G}}_{X}=\;{\text{E}}_{MM}+\;{\text{G}}_{Solv}-\;{\text{TS}}_{MM}$$

[where, X = > complex, protein or ligand]

$${\text{E}}_{MM}={\text{E}}_{bond}+\;{\text{E}}_{angle}+{\text{E}}_{tors}+\;{\text{E}}_{vdw}+\;{\text{E}}_{elec}$$

E_MM_ is the average molecular mechanical energy, G_Solv_ is the calculated solvation free energy and TS_MM_ is the solute entropy. We did not apply entropy penalty to any of free energy calculations, hence TS_MM_ was zero for our studies. E_bond_, E_angle_, E_tors_, E_vdw_ and E_elec_ are bond, angle, torsion, van der Waals and Coulombic energies respectively. The binding energy contribution per residue was also calculated using MMGB/PBSA method. Analysis was then performed to know which residues have more binding affinity with the ligand as opposed to the natural substrate.

## Results and Discussion

### Structure refinement and binding site of cPEPCK

Native cPEPCK structure obtained from PDB (PDB ID: 1KHG) had missing tertiary structure information for amino acid residues in the range of 465 to 472 (TAAAEHKG) and 547 to 548 (KA). A complete structure of protein is essential for accurate MD simulation studies and hence these missing segments were generated and the structure was refined using MODELLER software. Several structures of cPEPCK were available at PDB, like holo-enzyme, GDP and substrate bound conformation, and GTP analogue bound conformation. These PDB structures of cPEPCK were superimposed on each other to study the changes in conformation of active site residues due to binding of different ligands. The conformation of binding site residues showed no significant difference in all the three states of cPEPCK. Low resolution might be the probable cause of inability to capture the differences in active sites of these PDB structures. Hence, we relied on structural energy minimization method to induce the effect of change in the binding site conformation because of the presence of different ligands. A refined structure of cPEPCK was transformed into three binding state conformations: u_cPEPCK, GTP_cPEPCK and GDP_cPEPCK, by superimposition. Minimization of u_cPEPCK, GTP_cPEPCK and GDP_cPEPCK incorporated appropriate changes in binding site of cPEPCK in accordance with the type of ligand present there. Though the overall structures of c_cPEPCK, GTP_cPEPCK and GDP_cPEPCK remained unaffected, few binding site residues including His255, Lys281, Asp301, Val326, Arg427, and Phe476 rearranged their positions under the effect of cofactors (S1A Fig) in GTP_cPEPCK protein. Binding site residues Gly289, Lys290, Thr291, Phe530 and Asn533 were directly interacting with GTP via hydrogen bonds while His264, Pro285, Ser286, Cys288, Asn292, Gly334, Val335, Gly338, Arg436, Trp516, Phe517, Phe525, Trp527, Pro528, and Gly529 were the other residues stabilizing the binding of GTP (S1B Fig).

The prepared u_cPEPCK structure was verified for its stability by a 30 ns MD simulation run using AMBER suite. The residues lining the binding pocket rearranged their spatial conformation during the simulation run so as to acquire an energetically favored stable state. The root mean square deviation (RMSD) of the u_cPEPCK protein backbone with reference to the first frame was plotted for the entire simulation time (Fig 1B). The protein backbone deviated around 1.5Å from its initial structure in the first 5ns, thereafter making a strong fluctuation to 3.2Å it again stabilized to around 2.5Å which then persisted till the end of the simulation run. Being an experimentally solved protein structure, the structure was close to its stable folding conformation as indicated by the low RMSD value.

**Fig 2:**
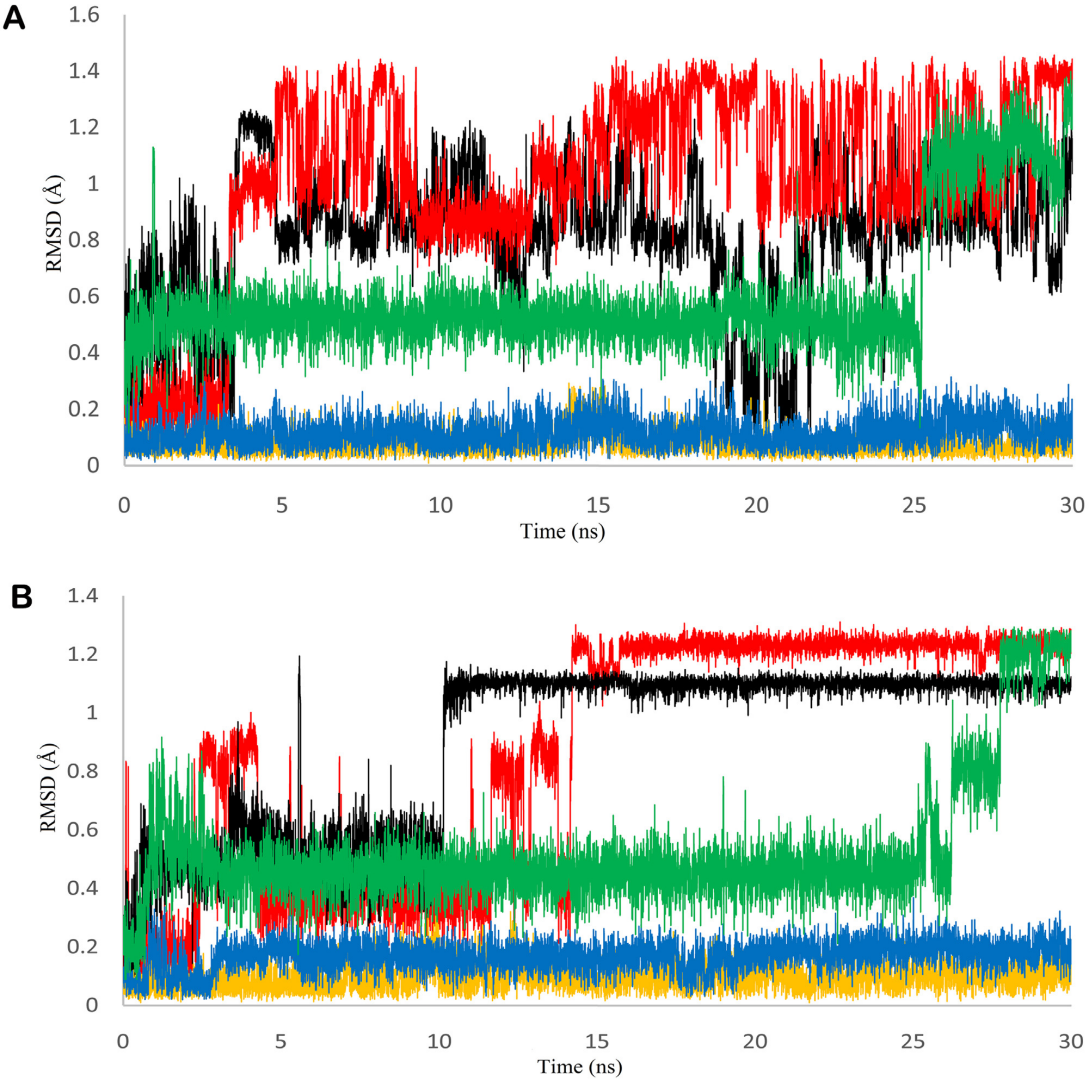
Fluctuation in residue Asn292 and Asp310 during the MD simulations. Changeability of GTP interacting residues indicates the change in position of ligand with respect to GTP binding site during the MD simulation. A) RMSD of residue Asn292 in refined cPEPCK protein backbone (black), backbone of u_cPEPCK-genistein complex (red), backbone of GTP_cPEPCK-genistein complex (blue), backbone of GDP_cPEPCK-genistein complex (yellow), and backbone of OAA-GTP_cPEPCK protein-ligand complex. B) RMSD of residue Asp310 in refined cPEPCK protein backbone (black), backbone of u_cPEPCK-genistein complex (red), backbone of GTP_cPEPCK-genistein complex (blue), backbone of GDP_cPEPCK-genistein complex (yellow), and backbone of OAA-GTP_cPEPCK protein-ligand complex.

Binding site of cPEPCK is a large cavity comprising of a GTP/GDP binding site and a substrate/product binding pocket (S2 Fig). Binding site of cPEPCK covers an area of 2754.15 Å^2^, out of which 1789.55 Å^2^ is hydrophilic and 350 Å^2^ is of hydrophobic in nature. High hydrophilic nature of the binding site is justified as it has to accommodate GDP/GTP cofactor that contains highly negatively charged phosphate groups. Such large binding pocket can accommodate a cofactor such as GTP that has surface area of approximately 400 Å^2^ and a ligand such as genistein that has surface area of approximately 250 Å^2^ in bound conformation. Fluctuations of binding site residues were recorded during the MD simulation of u_cPEPCK protein. Although most of the residues lining the binding pocket did not show much flexibility, Asn292 and Asp310 showed high fluctuations indicating that their side chains were free to move within the binding cavity (Fig 2A and 2B). These residues were located near the GTP/GDP binding site and play an important role by interacting with GTP/GDP. Binding site analysis of a cPEPCK structure from stable trajectory reveals that the binding site expanded and merged with a neighboring cavity, which was adjacent to GTP and substrate/product binding pocket. The area of this extended binding cavity becomes 4184.51 Å^2^, out of which 2865 Å^2^ forms the hydrophilic surface. Role of the adjacent cavity is described later in this study.

### Molecular interaction of genistein with different cPEPCK binding site conformations

Depending upon the catalysis mechanism of cPEPCK, genistein gets an opportunity to bind with three different conformations of the enzyme. Presence or absence of cofactor at active site affects the interaction of genistein with cPEPCK.

#### Molecular interaction of genistein with u_cPEPCK

**Fig 3:**
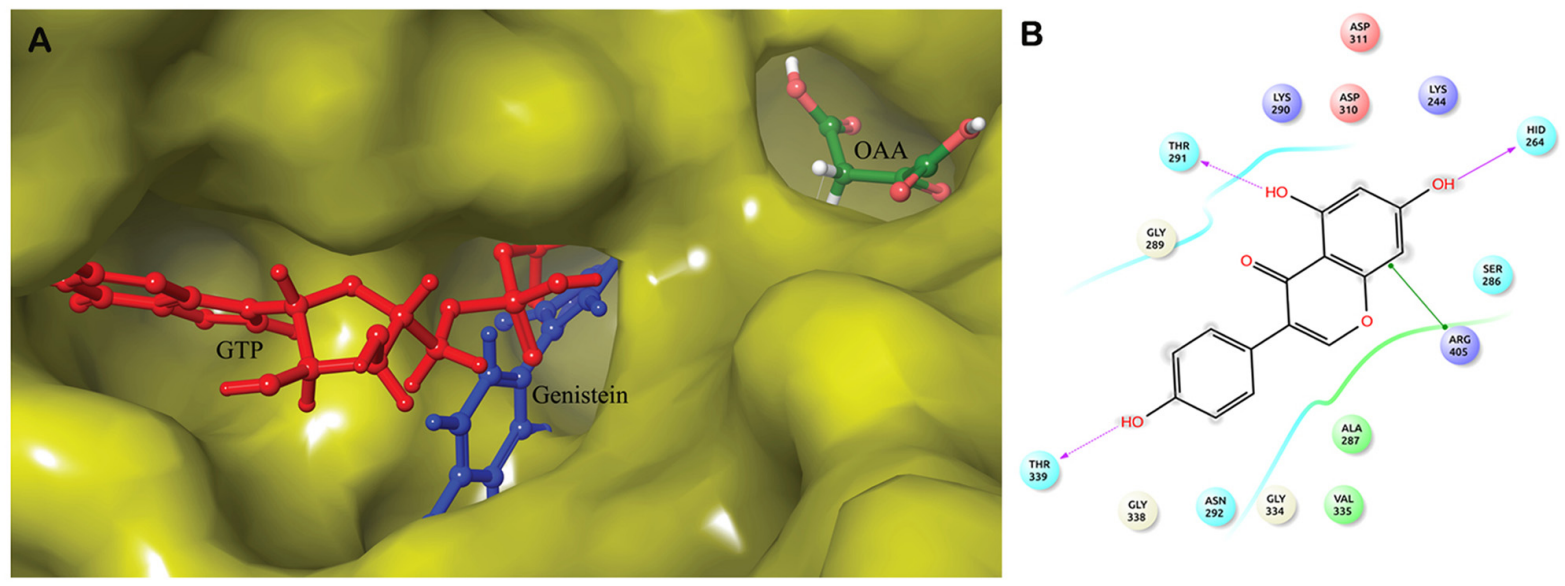
Binding of genistein with u_cPEPCK. A) Superimposed structure of u_cPEPCK-genistein complex and OAA-GTP_cPEPCK complex to identify location of genistein at binding site of u_cPEPCK after docking. Genistein (blue) can clearly be seen located at the site of GTP (red) binding. B) 2D representation of interaction of genistein with u_cPEPCK protein.

Absence of GTP or GDP at binding site in u_cPEPCK offers a larger cavity for ligand binding. Within large cavity only strong intermolecular interactions can hold a ligand stably. Hence, to assess the genistein binding with u_cPEPCK, prepared structure of genistein was docked with refined u_cPEPCK structure. Autodock score of -6.52 kcal/mol was observed indicating a strong affinity between the two molecules (Table 1). Semi-flexible docking methods do not incorporate the changes that take place in the protein structure due to ligand. Hence, to take into consideration the effect of genistein binding on the u_cPEPCK binding site, the docked structure was further refined by a ligand refinement web server PELE. Using the OPLS force field, the ligand binding refinement algorithm performed translations combined with small and large rotations of genistein within the binding site. Refinement generated the best docking pose of genistein within the binding site on u_cPEPCK, with RMSD of 6.62 Å in reference to the initial position of the ligand. The PELE binding score of -37.77 was obtained. When binding position of genistein was compared with GTP bound conformation of cPEPCK, it was found that genistein preferentially binds at GTP binding site in the absence of any co-factor at the binding site (Fig 3A). Genistein formed hydrogen bonds with Lys244, His264, Thr291, Tyr235, and Thr339 while hydrophobic interactions were seen with residues Ser286, Lys290 and Val335 (Table 2 and S3A Fig). Most of these residues were present within 4Å vicinity of genistein (Fig 3B). Ala287, Thr291, Lys290, and Val235 of u_cPEPCK involved in interaction with genistein were the residues that in general help in holding GTP at its binding site. Hence, it can be concluded that genistein in the absence of any cofactor preferentially binds to the GTP binding site of the enzyme.

**Table 1:** Docking scores and Binding free energies (ΔG) of protein-ligand complexes.

	u_cPEPCK (kcal/mol)	GDP_cPEPCK (kcal/mol)	GTP_cPEPCK (kcal/mol)
**Genistein**	**Docking score:** -6.52	**Docking score:** -6.24	**Docking score:** -5.82
	**MMGB/PBSA-ΔG (SD):** -18.36/-5.39 (± 2.56)	**MMGB/PBSA-ΔG (SD):** -22.61/-10.02 (± 2.96)	**MMGB/PBSA-ΔG (SD):** -23.26/-16.75/ (± 3.42)
**OAA**	-	-	**Docking score:** -4.38
**PEP**	-	**Docking score:** -6.85	-

**Table 2:** Interactions of cPEPCK with genistein, OAA and PEP.

Complexes	Pre MD Simulation	Post MD Simulation
**u_cPEPCK-Genistein**	**Hydrogen Bonds:** Lys244, His264, Thr291, Tyr235, Thr339	**Hydrogen Bonds:** Trp516
	**Hydrophobic:**Ser286, Lys290, Val335	**Hydrophobic:** Phe517, Phe530, Gly529
**GDP_cPEPCK-Genistein**	**Hydrogen Bonds:** Asp310, Lys290, Phe333	**Hydrogen Bonds:** None
	**Hydrophobic:** Lys243, His264, Ser298, Asp311	**Hydrophobic:** Gly289, Lys290, Gly334, Val335
**GTP_cPEPCK-Genistein**	**Hydrogen Bonds:** Tyr235, Lys244, His264, Asp311	**Hydrogen Bonds:** None
	**Hydrophobic:**Ala86, Ser286, Asn403	**Hydrophobic:** Glu86, Cys245
**GTP_cPEPCK-OAA**	**Hydrogen Bonds:** Arg87, Asn403, Arg405	**Hydrogen Bonds:** Thr92
	**Hydrophobic:** Tyr235	**Hydrophobic:** Cys245, Arg249
**GDP_cPEPCK-PEP**	**Hydrogen Bonds:** Arg87, Gly237, Asn403i), Arg405	-
	**Hydrophobic:** Ala86, Tyr235, Lys244, Phe485	

**Fig 4:**
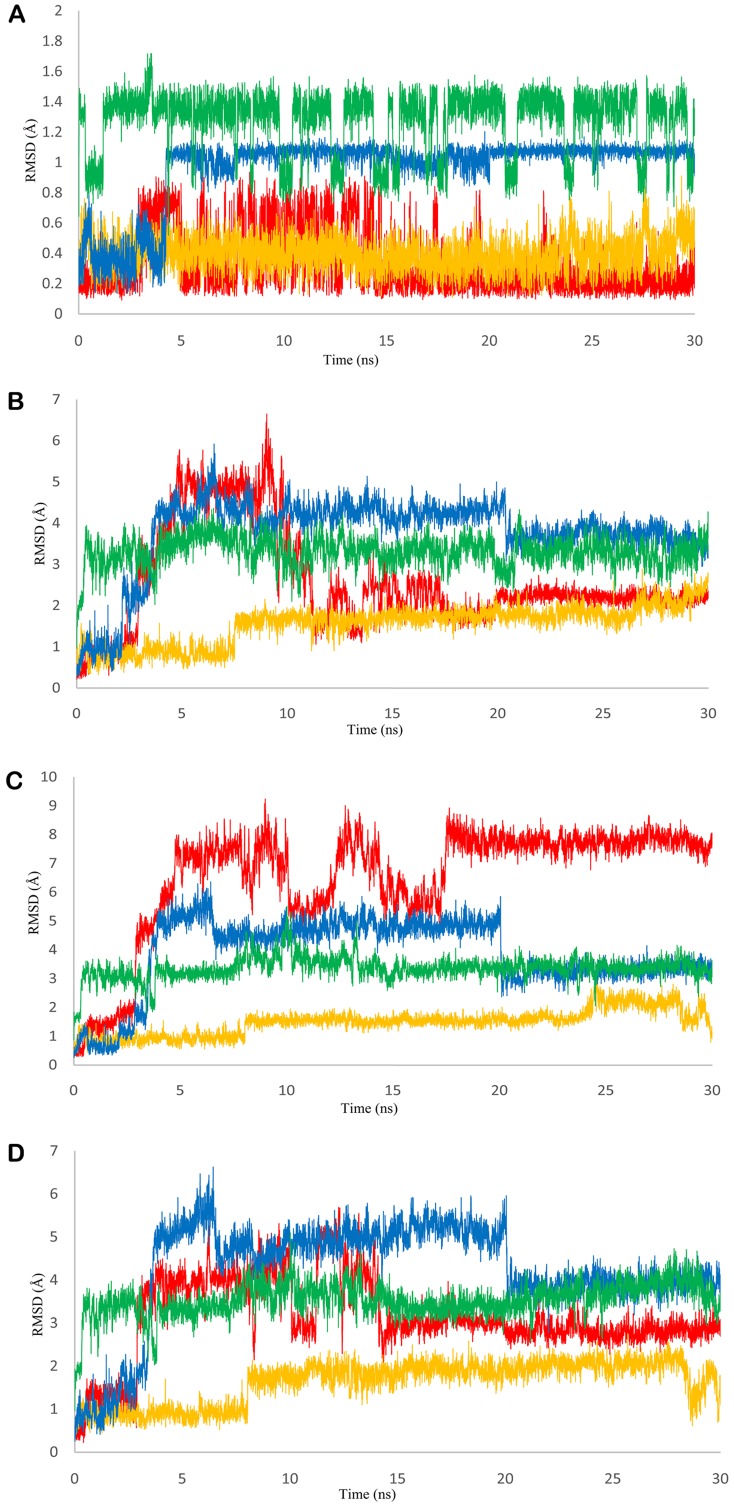
RMSD of PEPCK bound ligands and active site residues during MD simulations. A) RMSD graph of genistein in u_cPEPCK-genistein complex (red), GTP_cPEPCK-genistein complex (blue), GDP_cPEPCK-genistein complex (yellow), and OAA-GTP_cPEPCK protein-ligand complex (green). B) RMSD graph of genistein and residue Ala287 in u_cPEPCK-genistein complex (red), GTP_cPEPCK-genistein complex (blue), GDP_cPEPCK-genistein complex (yellow), and OAA-GTP_cPEPCK protein-ligand complex (green) C) RMSD graph of genistein and residue Phe333 in u_cPEPCK-genistein complex (red), GTP_cPEPCK-genistein complex (blue), GDP_cPEPCK-genistein complex (yellow), and OAA-GTP_cPEPCK protein-ligand complex (green) D) RMSD graph of genistein and residue Thr339 in u_cPEPCK-genistein complex (red), GTP_cPEPCK-genistein complex (blue), GDP_cPEPCK-genistein complex (yellow), and OAA-GTP_cPEPCK protein-ligand complex (green).

Further, to study the dynamics of genistein within the binding site of u_cPEPCK, we conducted 30 ns long MD simulation of u_cPEPCK-genistein complex. The protein backbone attained a stable conformation after 8 ns with RMSD value of around 2Å although the maximum fluctuation observed was about 2.5Å (Fig 1B). Within the binding pocket of u_cPEPCK, conformation of genistein did not change much during the simulation when compared to its initial conformation. This was evident from the low RMSD value of less than 1Å for the entire simulation run (Fig 4A). As, genistein prefers to bind at the GTP binding site of u_cPEPCK protein, its interaction analysis with the GTP interacting residues becomes important. Asn292, one of the GTP interacting residues, moved away from genistein after 4ns, displaying about 1Å fluctuation and thereafter remained stable till the end of simulation (Fig 2A). Similarly, Asp310 also deviated from its initial position between 11–13 ns interval but afterwards was found stable (Fig 2B). This data was in accordance to the MD simulation data of refined u_cPEPCK structure as Asn292 and Asp310 were found to be highly flexible residues of GTP binding site. Genistein was able to stabilize these residues to some extent. Binding site residues Ala287, Phe333 and Thr339 were stable and did not fluctuate much during the MD simulation run as inferred from the quite stable trajectory obtained after 15ns of simulation run (Fig 4B, 4C and 4D).

Though conformation of genistein did not change during the simulation, it moved within the large binding pocket of cPEPCK. Because of the movement of genistein within the binding site of cPEPCK, a slightly different interaction pattern was observed post simulation. The new interacting residues included Trp516, Phe517, Phe530 and Gly539, which were also the GTP interacting residues (S3B Fig). Although the number of hydrogen bonds decreased during the MD simulation, stable RMSD of genistein during the last 15 ns of MD simulation ensures its stability at GTP binding site. To identify the movement of genistein with respect to binding site residues, we calculated the distances between *center of mass* of genistein and the residues. Distance analysis revealed that genistein moved away from its initial binding location and shifted towards the guanosine binding region of GTP (Figures 1 and 2 in S1 Appendix). Binding free energy (ΔG) of genistein with u_cPEPCK as calculated by MMGB/PBSA method using the stable trajectory of MD simulations was -18.36/-5.39 kcal/mol (± 2.56) (Table 1). Here AutoDock score and ΔG value both affirm the binding of genistein at u_cPEPCK binding site. Though both the values, AutoDock score and ΔG depict an estimate of the binding free energy, the difference between their numerical value can be attributed to the different scoring functions that they use. AutoDock uses the entropy penalty while we have not incorporated its effect in MMGB/PBSA calculations. Also, in both these approaches the binding free energy score are relative rather than being absolute.

#### Molecular interaction of genistein with GTP_cPEPCK enzyme conformation

**Fig 5:**
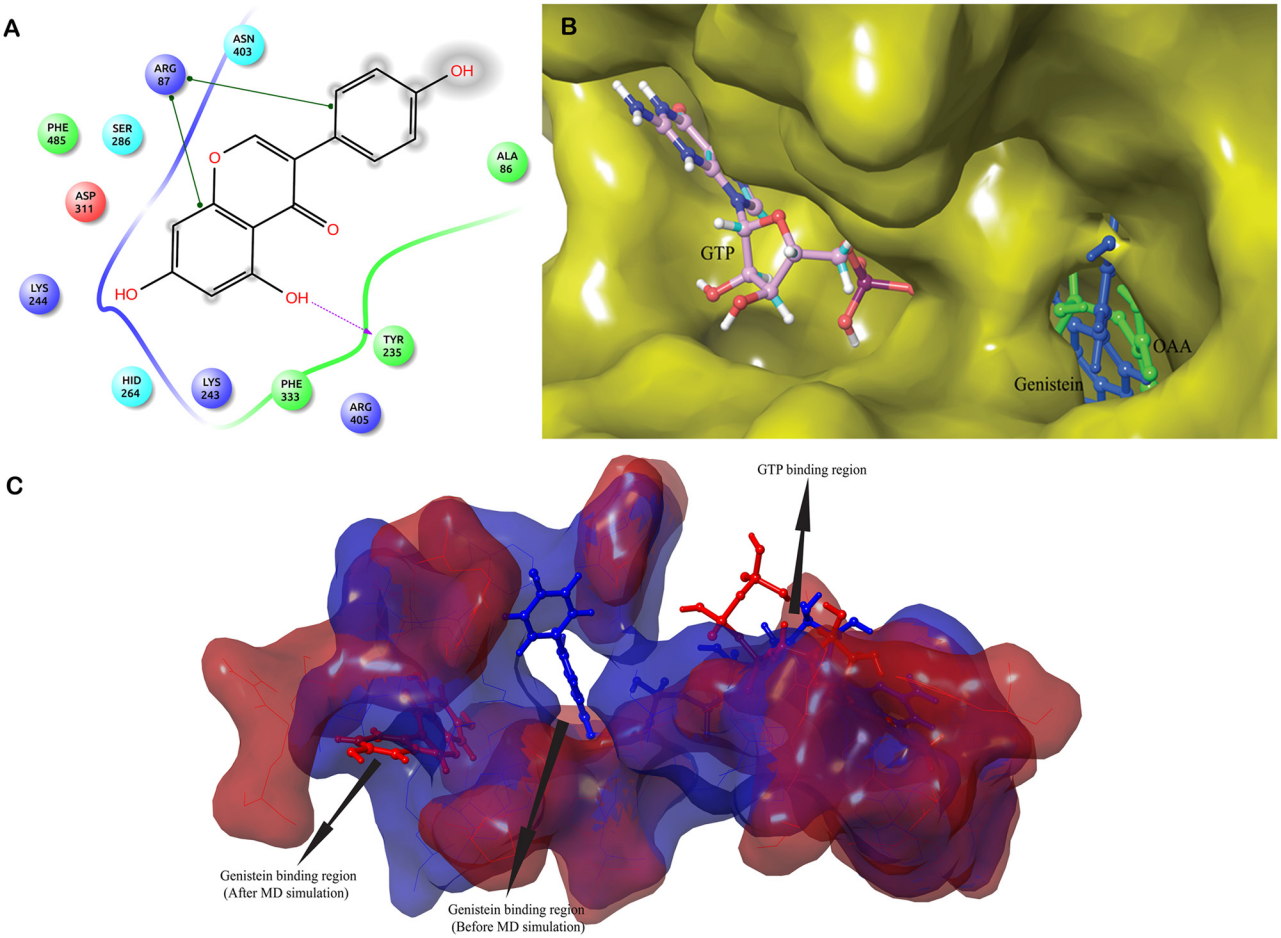
Binding of genistein with GTP_cPEPCK. A) 2D representation of interaction of genistein with GTP_cPEPCK protein. B) Superimposed structure of GTP_cPEPCK-genistein complex and OAA-GTP_cPEPCK complex to identify location of genistein at binding site of GTP_cPEPCK after docking. Genistein (blue) can clearly be seen located at the site of OAA (green) binding. C) Superimposed cavities of GTP_cPEPCK-genistein complex before the simulation (blue) and after the simulation (red). Change in the cavity shape and size can clearly be seen, therefore, genistein is rested in deferent conformation inside their respective cavities.

The conformation of cPEPCK active site changes with the binding of GTP. Presence of GTP created an environment similar to substrate bound enzyme for genistein binding. To evaluate the genistein binding with GTP_cPEPCK, prepared structure of genistein was docked with GTP_cPEPCK. Autodock docking score of -5.82 kcal/mol was observed indicating a strong affinity of genistein for GTP_cPEPCK (Table 1). Genistein formed hydrogen bonds with Arg87, Tyr235, Lys244, His264 and Asp311 and showed hydrophobic interactions with residues Ala86, Ser286 and Asp403 (Table 2, Fig 5A and S3C Fig). It was hence inferred that genistein binds at the substrate (OAA) binding site in the presence of GTP (Fig 5B).

To study the movements of genistein within the binding site of GTP_cPEPCK, we conducted 30 ns long MD simulation of GTP_cPEPCK-genistein complex. The maximum RMSD of protein backbone was found to be 2.5Å. After 3 ns of MD run, a stable conformation was achieved with RMSD of about 2Å (Fig 1B). Genistein changed its conformation after 4ns of simulation (Fig 4A). This altered conformation of genistein within GTP_cPEPCK was quite stable in the presence of GTP as can be seen by its RMSD of 1Å during the last 25 ns MD simulation. The interaction of genistein with GTP interacting residues was further analyzed. Ala287, Phe333 and Thr339 were stable and did not fluctuate much during the MD simulation. RMSD value of Ala287, Phe333 and Thr339 with genistein was almost stable after 4 ns simulation time with a drop in RMSD at 20 ns (Fig 4B, 4C and 4D). Interaction of Asn292 and Asp310 with genistein was highly stable during the simulation (Fig 2A and 2B). This high stability can be attributed to the presence of GTP at the binding site.

**Fig 6:**
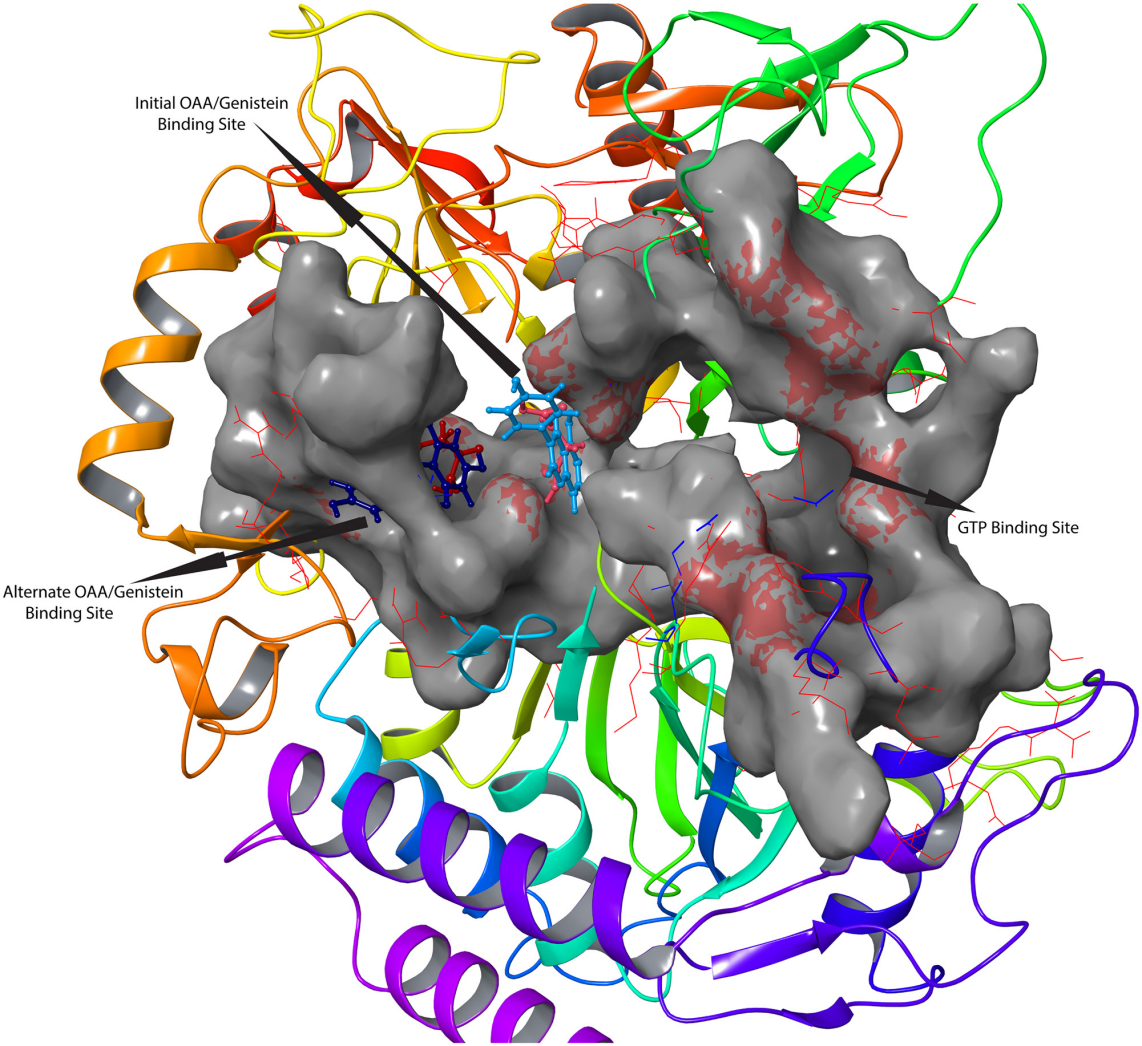
Change of binding site by both genistein and OAA after the MD simulations. Superimposed pre MD simulation and post MD simulation structures of OAA-GTP_cPEPCK and genistein-GTP_cPEPCK complexes, revealing the presence of possible binding sites within cPEPCK. Before the 30 ns MD simulation, OAA (light red) and genistein (light blue) are located at the same site while after the MD simulation OAA (dark red) and genistein (dark blue), both are located at the same extended binding site.

The changes in the conformation of active site in GTP_cPEPCK-gensitein complex before and after MD are shown in Fig 5C. Genistein was found trapped into an alternate small binding site located just adjacent to the main binding site (Fig 6). Glu86 and Cys245 were the residues interacting with genistein at the extended binding site of GTP_cPEPCK (Table 2 and S3D Fig). Also the distance analysis between genistein and Arg87, Tyr235, Arg249 suggested that in the presence of GTP, genistein stabilizes itself near the substrate/product binding site by moving into an extended binding site (Figure 3 in S1 Appendix). This indicates that behavior of genistein at the cPEPCK binding is different in the presence of GTP than that in unbound cPEPCK. In the presence of GTP, genistein stabilizes itself at the substrate/product binding site as opposed to moving towards GTP binding site in absence of any cofactor at cPEPCK binding site. Binding free energy (ΔG) of genistein with GTP_cPEPCK was estimated to be -23.26/-16.75 kcal/mol(±3.42) using MMGB/PBSA method (Table 1).

#### Molecular interactions of genistein with GDP_cPEPCK enzyme conformation

**Fig 7:**
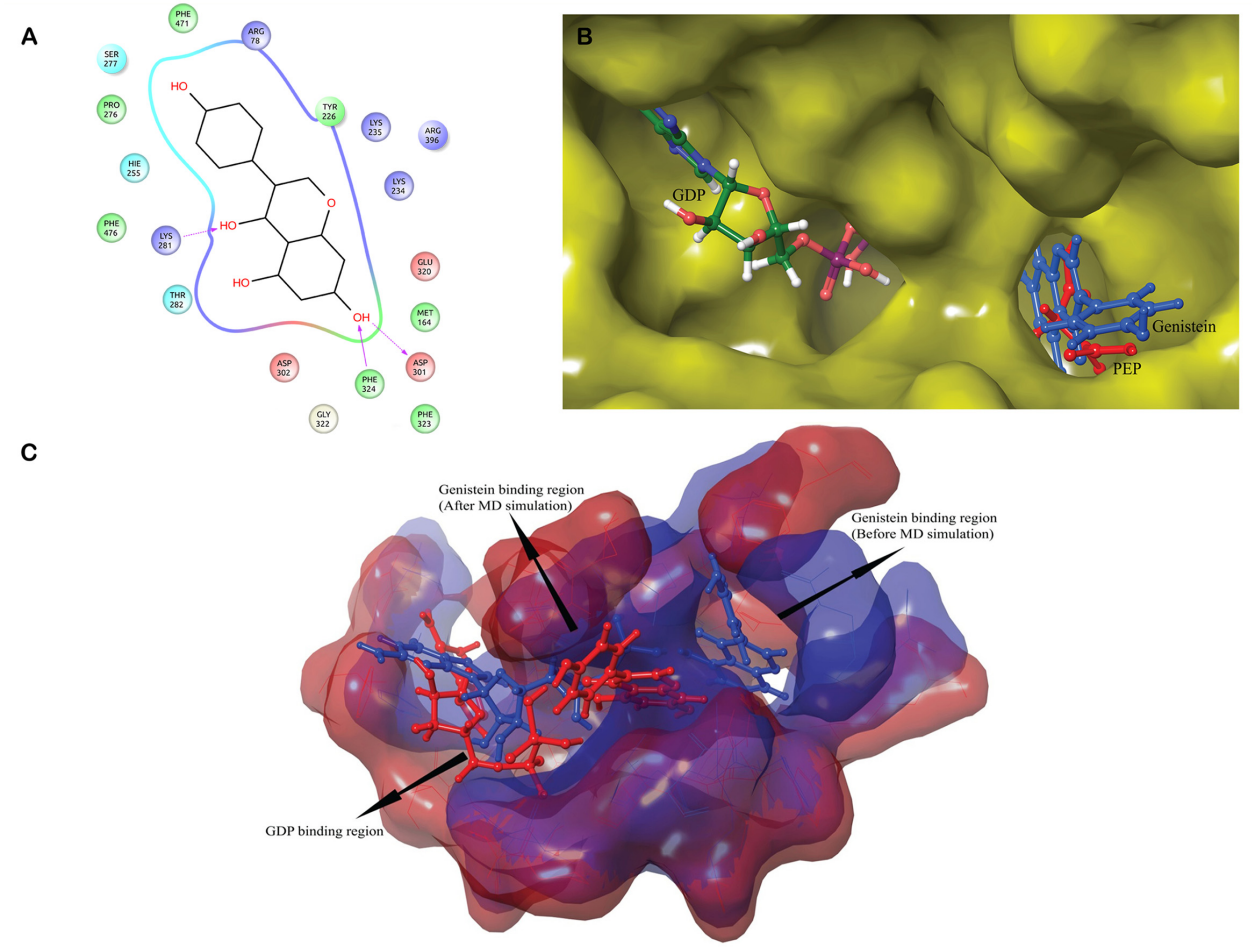
Binding of genistein with GDP_cPEPCK. A) 2D representation of interaction of genistein with GDP_cPEPCK protein. B) Superimposed structure of GTP_cPEPCK-genistein complex and OAA-GDP_cPEPCK complex to identify location of genistein at binding site of GTP_cPEPCK after docking. Genistein (blue) can clearly be seen located at the site of OAA (green) binding. C) Superimposed cavities of GDP_cPEPCK-genistein complex before the simulation (blue) and after the simulation (red). Change in the cavity shape and size can clearly be seen, therefore, genistein is rested in deferent conformation inside their respective cavities.

GDP_cPEPCK contains the GDP cofactor at binding site which provides an environment similar to the one that prevails after formation of product at the active site of cPEPCK naturally. To elucidate the molecular interactions of genistein with GDP_cPEPCK, prepared structure of genistein was docked with GDP_cPEPCK structure. Autodock docking score of -6.24 kcal/mol was observed indicating a strong affinity of genistein with cPEPCK (Table 1). Genistein was found forming hydrogen bonds with Lys290, Asp310, Phe333 and Arg405 while it was forming hydrophobic contacts with Lys243, His264, Ser298 and Asp311 (Fig 7A and S3E Fig). Analysis of all the residues of GDP_cPEPCK involved in genistein binding revealed that in the presence of GDP, genistein binds at product (PEP) binding site (Fig 7B).

To further study the dynamic behavior of genistein within the binding site of GDP_cPEPCK, 30 ns long MD simulation of GDP_cPEPCK-genistein complex was performed. Backbone of the protein deviated approximately 2Å from the initial conformation in the first 3ns after which the conformation was quite stable and persisted till the end of the simulation run (Fig 1B). Genistein was also stable at its binding site throughout the MD simulation as indicated by the RMSD value of less than 1Å (Fig 4A). Ala287, Phe333, and Thr339 in genistein bound state did not show much fluctuation (RMSD ~1Å) (Fig 4B, 4C and 4D). Asn292 and Asp310 were also highly stable due to the presence of GDP at the binding site (Fig 2A and 2B). Changes in the structure of GDP_cPEPCK because of genistein binding can be seen in Fig 7C. It was obtained by superimposing the first and the last frame of the simulation run. Post simulation, genistein was now interacting with Gly289, Lys290, Gly334, and Val33 (Table 2 and S3F Fig). Genistein kept moving away from the Arg249 throughout the simulation and moved almost 5Å away from its initial binding site at the end of the simulation (Figure 4 in S1 Appendix). These results suggest that in the presence of GDP, genistein shifts away from the substrate/product binding site but does not move to extended binding site. Binding free energy (ΔG) of genistein with GDP_cPEPCK using MMGB/PBSA method was calculated to be -22.61/-10.02 kcal/mol (±2.96) for the stable simulation trajectory (Table 1).

### Interaction of natural substrate OAA and product PEP with cPEPCK

Natural substrate OAA and product PEP of cPEPCK were also docked to cPEPCK to compare them with the docking results of genistein. Substrate OAA was docked with GTP_cPEPCK while PEP was re-scored with GDP_cPEPCK. Docking score of OAA with GTP_cPEPCK was -438 kcal/mol and it was interacting with residues Arg87, Asn403, and Arg405 via hydrogen bonds and Tyr235 via hydrophobic interactions (S3G Fig). The docking score of PEP with GDP_cPEPCK was -6.85 kcal/mol (for the experimentally solved pose). PEP was interacting with Arg87, Gly237, Asn403, and Arg405 via hydrogen bonds (Tables 1 and 2, S3H Fig).

Stability of GTP_cPEPCK-OAA complex was also examined by 30 ns long MD simulation. Protein backbone of GTP_cPEPCK was stable like the backbone of cPEPCK during other simulations (Fig 1B). RMSD of OAA within GTP_cPEPCK during the 30 ns MD was found fluctuating between 1.0 Å and 1.4 Å (Fig 4A). It was observed that just after the start of simulation, OAA shifts towards the extended binding site adjacent to the substrate binding site (Fig 6) and keeps fluctuating between the two sites as indicated by the RMSD plot (Fig 4A). OAA was interacting with Thr92, Cys245 and Arg249 residue of GTP_cPEPCK after the completion of 30 ns MD simulation (S3I Fig). These residues were part of an extended binding site (Table 2). During this simulation, distance analysis between OAA and Arg87 & Arg249 confirms that OAA moved to the extended binding site just after initial 500ps of simulation and stabilized itself at this site (Figure 5 in S1 Appendix).

Also, careful analysis of GTP_cPEPCK-OAA complex after docking and during the MD simulation revealed that the extended binding site was guarded by a basic residue, Arg87 while another arginine residue at 249^th^ position was a part of this cavity. Initially in docked GTP_cPEPCK-OAA complex, Arg87 was closing the entry of the extended binding site while during the simulation; side chain of Arg87 moved a side; opening the gate of the extended binding site. Similarly, Arg249 was present within the extended site in experimental structure (during docking). As Arg87 moved to open the extended site, Arg249 also changed its position to make extended site unoccupied (S4 Fig). After the opening of the extended site, OAA was able to enter into it. Hence, one can suggest that Arg87 plays the role of “Gatekeeping” residue for the extended binding site. Once the gate was opened, it did not close during the entire MD simulation process. Probably, the closing of the cavity by Arg87 needs a vacant site which is possible only after the release of product.

### Extended binding sites in cPEPCK_OAA and Genistein_cPEPCK complexes

Comparison of extended binding site in OAA-GTP_cPEPCK and genistein-GTP_cPEPCK complex revealed that both are the same sites (Fig 6). OAA and genistein were interacting with the same residues lining the extended binding site (Table 2). Both OAA and genistein were binding to this site of cPEPCK in the presence of GTP. It was evident that genistein did not bind at the extended site in the presence of GDP or in unbound PEPCK form. These results indicated that the presence of GTP is important for binding of substrate/inhibitor at the extended binding site and binding at the extended site is important for better stability of substrate/inhibitor. Shifting of OAA at the extended site could be a critical event for the transfer of a phosphate group from GTP because OAA earns extra space to accommodate the phosphate by shifting into the extended site.

Extended binding site is comprised of residues Arg87, Arg249, Pro475, and Phe476, among which Arg249 remained almost static. Analysis of all the simulation trajectories revealed the importance of residue Arg87 in regulating the movement of bound substrate or inhibitor. Arg87 was located such that it acted as a gatekeeping residue for the extended binding site and could also interact with Glu460 of a loop consisting of residues from 465 to 474 (loop 465–474). During all the simulation trajectories, loop 465–474 was highly mobile, except while interacting with Arg87. The distance analysis between COM of loop 465–474, Arg87, and genistein/OAA allowed us to correlate the movement of substrate/inhibitor at the binding site and movement of loop 465–474. According to the distance analysis results in u_cPEPCK, genistein shifted at GTP binding site that was at equal distance from the loop and its initial position (Figure 6 in S1 Appendix). In GTP_cPEPCK, genistein moved towards extended binding site but in GDP_cPEPCK, like u_cPEPCK, genistein again moved away from the extended binding site (Figure 6 in S1 Appendix). Also, the closest association between Arg87 and loop 465–474 was observed when OAA was bound with cPEPCK.

To see the effect of genistein on the extended binding site, we measured the volume of the extended binding site during the simulation using GTP_cPEPCK-genistein complex. The volume of the extended binding site around the genistein gradually increased from ~1437Å^3^ to 2547 Å^3^, almost 1.8 times of its initial volume (S5 Fig). The hydrophobic contribution in increase of area of extended binding site was very low as compared to hydrophilic contribution. This indicated that the extended binding site comprised mostly of hydrophilic residues.

### Binding of 3 beta-mercaptopicolinic acid and genistein at extended site of cPEPCK

3-mercaptopicolinic acid (3-MPA) is a well-known non-competitive inhibitor of cPEPCK [63]. Non-competitive inhibition of cPEPCK by 3-MPA suggests that it binds at a site other than the product/substrate binding site. To check the binding mode of 3-MPA and role of extended binding site, we docked 3-MPA at the extended site of cPEPCK in the presence of GTP and OAA. 3-MPA was able to bind at the extended site in the presence of substrate and GTP. These results are in accordance with the fact that 3-MPA inhibits cPEPCK non-competitively without hindering the binding of OAA. Stability of 3-MPA at the extended site in the presence of OAA was also studied by 45ns MD simulation. Protein backbone was highly stable during the MD simulation, ensuring that protein did not change its native conformation when 3-MPA was docked to cPEPCK (S6A Fig). 3-MPA itself was highly stable at the extended site and showed only a 1Å RMSD deviation occasionally (S6B Fig). However, 45ns MD simulation revealed that OAA became destabilized as 3-MPA occupied the extended site. OAA was highly unstable during the entire MD simulation and almost popped out of cPEPCK’s binding cavity in several frames (S6C Fig). However, its complete exit was prevented by phosphate tail of GTP (S7A Fig).

As genistein was found to bind at the extended site during the MD simulations, we also docked genistein at the extended site in the presence of OAA. Like 3-MPA, genistein was also able to dock at the extended site in the presence of cPEPCK’s natural substrate, OAA. This suggests that genistein may also follow the same strategy of non-competitive inhibition like 3-MPA. Further genistein’s stability at the extended site in the presence of substrate was confirmed by 50ns MD simulation run. Like other simulations involving cPEPCK, protein backbone was highly stable during the MD simulation (S6A Fig). Genistein occupied the extended site for most of the time during the 50ns MD simulation, with occasionally shifting its phenol tail to substrate binding site (S6B Fig). However, during the simulation, OAA slowly moved out of the binding pocket just after 10 ns simulation time and left the cPEPCK binding pocket (S6C and S7B Figs). Release of OAA occurred after phosphate tail of GTP shifted outwards and the surrounding loops (comprised of residues 85–91 and residues 465–474) changed their conformation during the simulation making a way for OAA to come out. Such attempts of leaving cPEPCK binding site were hindered by phosphate tail of GTP when 3-MPA was bound to cPEPCK.

We measured the distances between the COM of genistein, OAA and binding site residues to track the movement of OAA and genistein during the simulation. Changes in distance clearly showed the release of OAA from binding pocket, in the presence of genistein at the substrate binding site and also the stability of genistein at the same time (Figure 7 in S1 Appendix). There were also indications of the slight shifting of genistein out of extended binding site (Figure 8 in S1 Appendix). This result is in accordance with the observation that genistein was shifting between extended binding site (initial binding site for this case) and the substrate binding site. The shifting occurred only when OAA had left the substrate binding site. These results further strengthen the possibility of genistein being a potential inhibitor of cPEPCK and shed light on its inhibition mechanism.

### Possible role of the extended binding site in cPEPCK

Based on the findings of our study, there seems to be a definite role of extended binding site in cPEPCK. Existence and functional role of this extended site has not been studied so far. Not only extended site seems to play an important role in cPEPCK’s catalytic mechanism, non-competitive inhibitors like 3-MPA also seem to exploit this site to inhibit the functional activity of cPEPCK. Initially the opening of site is blocked by Arg87 and the site remains occupied by Arg249. The movement of Arg87 opens the extended site, making extra room to displace further away from GTP (S4 Fig). What triggers the Arg87 to move and open the extended site is still to be explored, but presence of GTP at binding site seems to be an essential criterion. Also, our study noted a relation between the movement of Arg87, Arg249, R-loop and omega-loop of cPEPCK. R and omega-loop (referred as loop 465–474 in this study) have been reported to regulate the catalysis of OAA in cPEPCK [64]. However, the same site can also be exploited by other molecules to inhibit the function of cPEPCK as 3-MPA and genistein were found to do in this study.

## Conclusions

In this study, we generated three possible conformations of cPEPCK based on the presence or absence of cofactors at the binding site. All three conformations were used to check the binding mode of genistein with cPEPCK. Binding of genistein with cPEPCK was also compared with cPEPCK’s natural substrate OAA and product PEP. Initially genistein binds to cPEPCK with almost same binding affinity in all three conformations namely, u_cPEPCK, GTP_cPEPCK and GDP_cPEPCK but MD simulations revealed the fate of genistein within their corresponding binding sites. In the absence of any cofactor, genistein preferentially binds at the GTP binding site while presence of GTP or GDP affected the binding of genistein in different manners. In the presence of GTP, genistein tends to shift towards an extended binding site and is highly stable at that site. This extended binding site of genistein was also the extended binding site for OAA. In GDP bound conformation of cPEPCK, genistein stayed near the substrate binding site but it was not as stable as it was in GTP bound state.

Our study also revealed that the presence of GTP leads to the binding of substrate/inhibitor at the extended binding site. Also substrate/inhibitor bound at the extended binding site forms the most stable conformations. This study demonstrates that the natural substrate of cPEPCK also uses the extended binding site, at which, genistein binds strongly in the presence of GTP. Also, one of the known non-competitive inhibitor of cPEPCK, 3-MPA seems to exploit the extended binding site to inhibit cPEPCK non-competitively. Furthermore, our study proves that if a ligand (3MPA and genistein), binds at the extended binding site, still the natural substrate of cPEPCK, OAA, can bind at the substrate binding site, facilitating the non-competitive inhibition mechanism.

To conclude, genistein showed potential to bind at the GTP and product binding sites, though the binding was not highly stable, it may inhibit cPEPCK competitively against GTP or PEP. However, highly stable binding of genistein at the extended binding site, similar to that of OAA and 3-MPA, strongly suggests that it might inhibit cPEPCK non-competitively. Hence, genistein seems to inhibit cPEPCK by the mechanism of mixed inhibition, which includes both competitive and non-competitive inhibition methods.

## Supporting Information

S1 Appendix Distance analysis between ligands and binding site residues of various conformations of cPEPCK. (DOCX)

S1 Fig GTP binding site of cPEPCK. A) u_cPEPCK (green), GTP_cPEPCK (red) and GDP_cPEPCK (blue) are superimposed on each other to depict the changes in GTP binding site residues after energy minimization. B) 2D representation of interactions between GTP and its binding site residues in cPEPCK.
(TIFF)

S2 Fig Structure of cPEPCK illustrating the GTP binding site and substrate/product binding site. (TIFF)

S3 Fig Ligplot interaction diagrams between cPEPCK protein and its ligands. A) Interactions between u_cPEPCK and genistein before the 30 ns MD simulation. B) Interactions between u_cPEPCK and genistein after the 30 ns MD simulation. C) Interactions between GTP_cPEPCK and genistein before the 30 ns MD simulation. D) Interactions between GTP_cPEPCK and genistein after the 30 ns MD simulation. E) Interactions between GDP_cPEPCK and genistein before the 30 ns MD simulation. F) Interactions between GDP_cPEPCK and genistein after the 30 ns MD simulation. G) Interactions between GTP_cPEPCK and OAA before the 30 ns MD simulation. H) Interactions between GTP_cPEPCK and OAA after the 30 ns MD simulation. I) Interactions between GTP_cPEPCK and PEP after molecular docking.
(TIFF)

S4 Fig Movement of Arg87 and Arg249 during MD simulation of OAA_cPEPCK complex. Initially when OAA is binding at substrate binding site, Arg87 has blocked the entrance of extended binding site while Arg249 has occupied the extended site (Red/Brown colored). During the simulation, Arg87 moved aside and Arg249 has also changed its conformations and OAA has shifted to the extended site (Green colored).
(TIFF)

S5 Fig Change in the area of extended binding site around genistein during the simulation of GTP-cPEPCK and genistein complex. Black bars represent the total area, green represents the hydrophilic area and orange indicates the hydrophobic area. The first bar is for the area of extended site of the docked genistein GTP_cPEPCK complex. Genistein was docked at the substrate binding site and the area was calculated for the available binding space around 6Å of genistein. Later, as the simulation proceeded and genistein started moving into the extended binding site, its area also increased. Also, as the total area increased, hydrophilic area also increased.
(TIFF)

S6 Fig RMSD graphs during MD simulations of 3-MPK and genistein at extended sites in the presence of OAA. A) RMSD in cPEPCK backbone in the presence of 3-MPA (Red) and genistein (Blue) at extended sites. B) RMSD of 3-MPA (Red) and genistein (Blue) during their MD simulation at extended sites. Genistein did not move after it was stabilized at 2ns simulation time. 3-MPA is having less RMSD than genistein but it tends to change its conformation at intervals. C) Unstable OAA in the presence of 3-MPA or genistein at the extended site is displaying very high RMSD values. In the presence of 3-MPA (Red), OAA was moving within the binding site but could not leave the binding site while in the presence of Genistein (Blue), OAA left the binding site at 10ns simulation time.
(TIFF)

S7 Fig Superimposed frames of cPEPCK before the MD simulation and after the MD simulation in the presence of 3-MPA and genistein at the extended site. A) Initially OAA is present at its binding site and 3-MPA is bound adjacent to it (Cyan carbon skeleton). But during 45ns MD simulation, unstable OAA is trying to exit the cPEPCK binding site while 3-MPA still sits at the same extended pocket. B) Genistein is docked at the extended binding site, adjacent to OAA (Blue colored carbon skeleton). But during its 50ns MD simulation, unstable OAA actually has left the binding pocket of cPEPCK while genistein is still present at the extended site (Cyan colored carbon skeleton). Genistein’s phenol tail has changed its position and has shifted to OAA binding site.
(TIFF)
