# Supplementary material for: Mixed Inhibition of cPEPCK by Genistein, Using an Extended Binding Site Located Adjacent to Its Catalytic Cleft
Source: PLoS One. 2015 Nov 3;10(11):e0141987. doi: 10.1371/journal.pone.0141987 (PMC4631375; doi:10.1371/journal.pone.0141987)
Supplement: S1 Appendix — (DOCX) [file pone.0141987.s001.docx]

**Mixed inhibition of cPEPCK by genistein, using an extended binding site located adjacent to its catalytic cleft**

Shashank P. Katiyar, Arpit Jain, Jaspreet Kaur Dhanjal and Durai Sundar

Department of Biochemical Engineering and Biotechnology, Indian Institute of Technology (IIT) Delhi, Hauz Khas, New Delhi 110016, India

**S1 Appendix**

| **Figure No.** | **Distance between centre of mass of A and B as given below** | | **Complex studied** | |
| --- | --- | --- | --- | --- |
|  | **A** | **B** | **Protein** | **Ligand** |
| S1 Appendix-Figure 1 | Genistein | cPEPCK substrate binding site | u_cPEPCK | Genistein |
| S1 Appendix-Figure 2 | Genistein | cPEPCK GTP binding site | u_cPEPCK | Genistein |
| S1 Appendix-Figure 3 | Genistein | cPEPCK substrate binding site | GTP_cPEPCK | Genistein |
| S1 Appendix-Figure 4 | Genistein | cPEPCK substrate binding site | GDP_cPEPCK | Genistein |
| S1 Appendix-Figure 5 | OAA | cPEPCK substrate binding site | GTP_cPEPCK | OAA |
| S1 Appendix-Figure 6 | Arg87/Ligand | Loop 465-474 | All complexes | |
| S1 Appendix-Figure 7 | OAA | cPEPCK substrate binding site | GTP_OAA_cPEPCK | Genistein |
| S1 Appendix-Figure 8 | Genistein | cPEPCK substrate binding site | GTP_OAA_cPEPCK | Genistein |


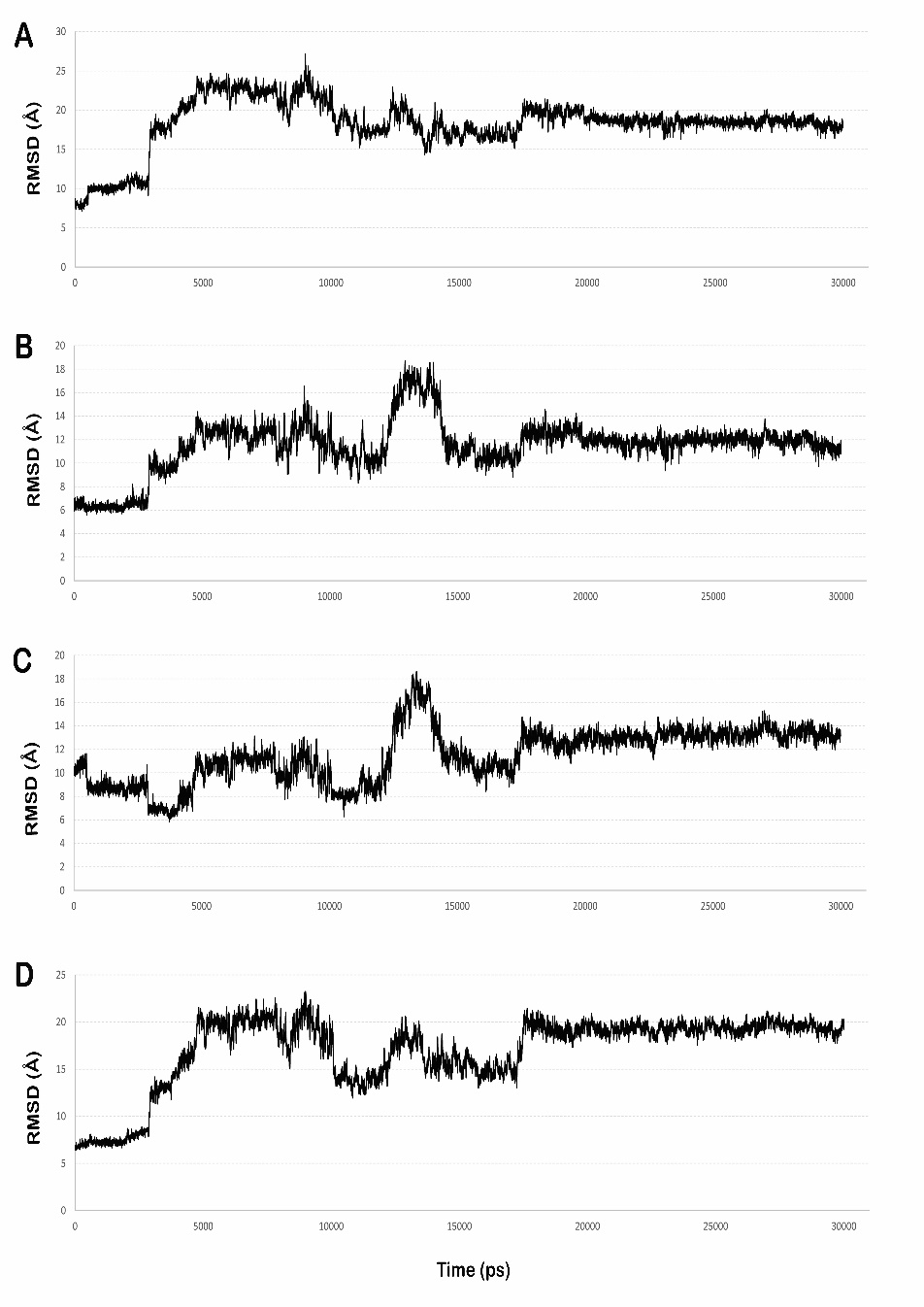
**S1 Appendix-Figure 1: Fluctuation in distance (Å) between centre of mass (COM) of genistein and cPEPCK substrate binding site residues during simulation of u_cPEPCK and genistein complex.** A) Change in distance between the COM of genistein and COM of His264. B) Change in distance between the COM of genistein and COM of Thr291. C) Change in distance between the COM of genistein and COM of Thr339. D) Change in distance between the COM of genistein and COM of Arg405.

It was observed that Arg87, His264, Thr339, Thr291 and Arg405 are the residues to which genistein was binding initially, and Gly289, Lys290, Thr291, Phe530 and Asn533 were the residues located near GTP binding site. While Gly289, Lys290 and Thr291 interacted with phosphate groups of GTP, Phe530 and Asn533 interacted with guanosine of GTP. Hence, Arg87, His264, Thr339, Thr291, Arg406 were closest to the initial binding location of genistein, followed by Gly289, Lys290, Thr291 and farthest were Phe530 and Asn533. COM of genistein moves away from His264, Thr291, Thr339 and Arg405 by approximately 10Å, 6Å, 3Å, and 11Å respectively.


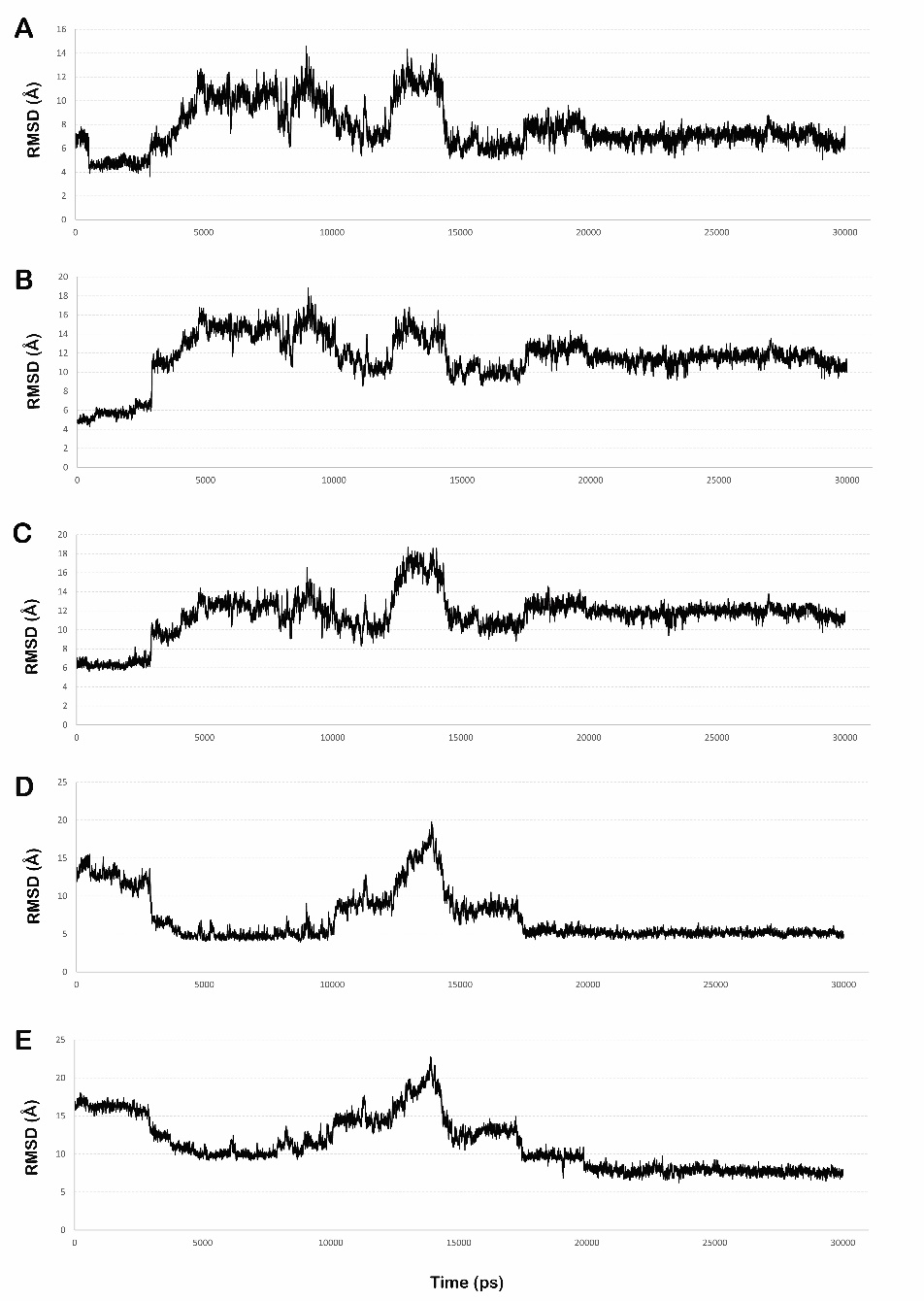
**S1 Appendix-Figure 2: Fluctuation in distance (Å) between COM of genistein and cPEPCK GTP binding site residues during simulation of u_cPEPCK and genistein complex.** A) Change in distance between the COM of genistein and COM of Gly289. B) Change in distance between the COM of genistein and COM of Lys290. C) Change in distance between the COM of Phe530 E) Change in distance between the COM of genistein and COM of Asn533.

COM of genistein also moved away from phosphate binding residues of GTP such as Gly289, Lys290, Thr291 by approximately 2Å, 7Å, and 6Å respectively. However, COM of genistein moved closer to the guanosine binding residues of GTP, Phe530 and Asn533 by 10Å and 9Å respectively.

**S1 Appendix-Figure 3: Fluctuation in distance (Å) between COM of genistein and cPEPCK substrate binding site residues during simulation of GTP_cPEPCK and genistein complex.** A) Change in distance between the COM of genistein and COM of Arg87. B) Change in distance between the COM of genistein and COM of Arg249. C) Change in distance between the COM of genistein and COM of Tyr235.


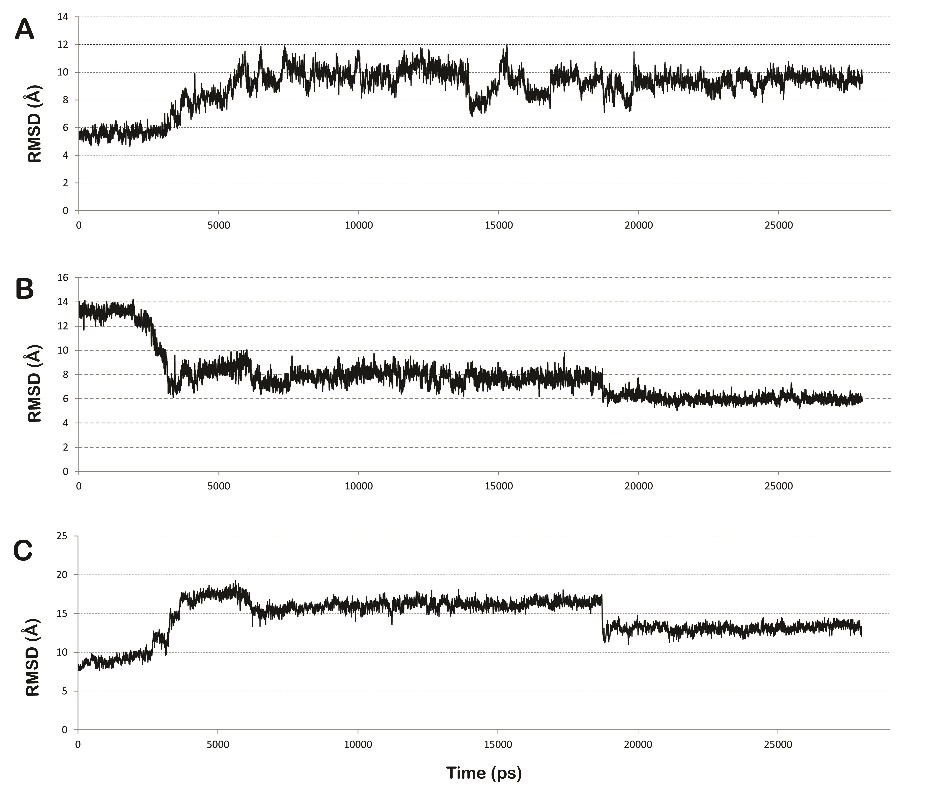
To track the movement of genistein at the binding site of GTP_cPEPCK, we performed the distance analysis between COM of substrate binding residues and COM of genistein. The distances were measure between COM of Arg87, Tyr235, Arg249 and COM of genistein. Arg87 and Tyr235 were interacting via hydrogen bonds with the initial binding mode of genistein while Arg249 is a stable residue of the extended binding site which can be used as an anchor to track the movement of genistein. COM of the genistein moves away by approximately 4 Å from COM of both Arg87 and Tyr235 at the end of the simulation. But COM of genistein came closer to the COM of the extended binding site residue Arg249 by approximately 5Å.

**S1 Appendix-Figure 4: Fluctuation in distance (Å) between COM of genistein and cPEPCK substrate binding site residues during simulation of GDP_cPEPCK and genistein complex.** A) Change in distance between the COM of genistein and COM of Arg87. B) Change in distance between the COM of genistein and COM of Arg249. C) Change in distance between the COM of genistein and COM of Arg405. D) Change in distance between the COM of genistein and COM of Lys290.


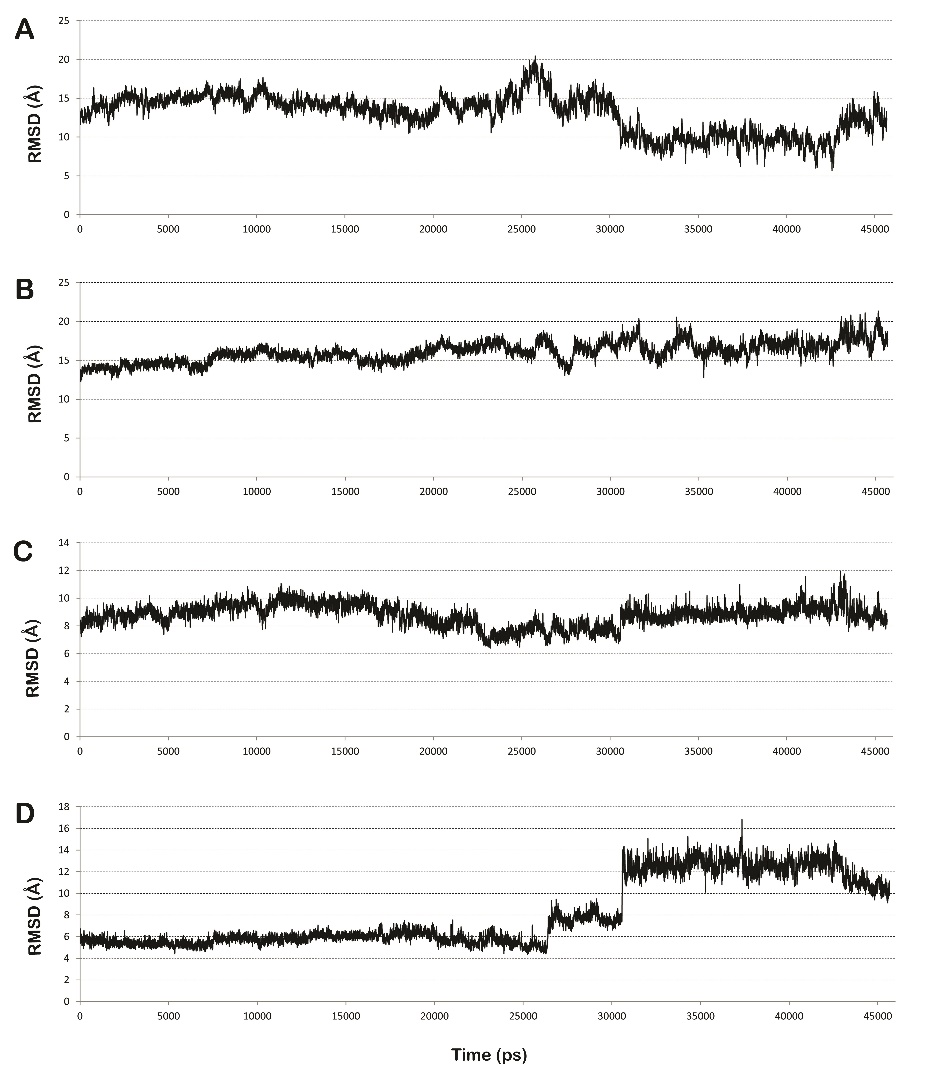
To track the movement of genistein within the binding pocket of GDP bound cPEPCK, distances were calculated between the COM of genistein and COM of genistein interacting residues in its initial binding state. While Arg87, Arg405, and Lys290 were interacting via hydrogen bonds with genistein, Arg249 was a stable residue of extended binding site. Hence, Arg87, Arg249, Lys290, and Arg405 were the most appropriate residues to track the movement of genistein. Genistein remained closed to Arg87 and Arg405 at the end of the simulation while its COM moved away from COM of Lys290 by approximately 4 to 5 Å.

**S1 Appendix-Figure 5: Fluctuation in distance (Å) between COM of OAA and cPEPCK substrate binding site residues during simulation of GTP_cPEPCK and OAA complex.** A) Change in distance between the COM of genistein and COM of Arg87. B) Change in distance between the COM of genistein and COM of Arg249.

To further validate the movement of OAA within the binding site of cPEPCK, changes in distances between COM of OAA and COM of OAA binding residues were measured. The
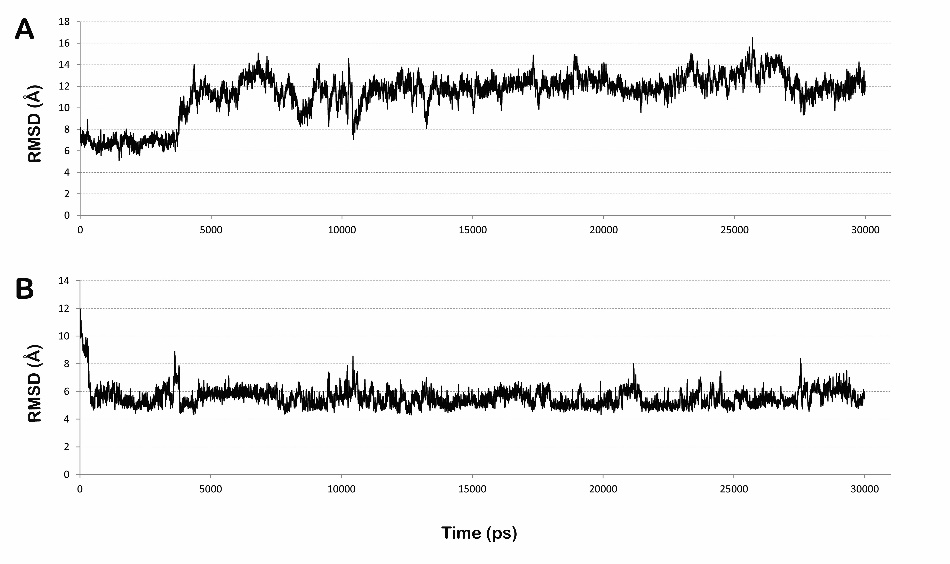
distance between OAA and Arg87 increased by 2Å to 4Å during the simulation, indicating either OAA moved away from Arg87 or vice versa. Distance analysis between OAA and Arg249 indicated that the distance decreases by 6Å very quickly, within 500 to 600 ps of simulation time.

**S1 Appendix-Figure 6: Fluctuation in distance (Å) between COMs of Arg87/ligand and COM of loop 465-474 during various simulation studies.** A) Change in distance between the COM of loop 465-474 and COM of Arg87 in u_cPEPCK and genistein complex simulation trajectory. B) Change in distance between the COM of loop 465-474 and COM of genistein in u_cPEPCK and genistein complex simulation trajectory. C) Change in distance between the COM of loop 465-474 and COM of Arg87 in GTP_cPEPCK and genistein complex simulation trajectory. D) Change in distance between the COM of loop 465-474 and COM of genistein in GTP_cPEPCK and genistein complex simulation trajectory. E) Change in distance between the COM of loop 465-474 and COM of Arg87 in GDP_cPEPCK and genistein complex simulation trajectory. F) Change in distance between the COM of loop 465-474 and COM of genistein in GDP_cPEPCK and genistein complex simulation trajectory. G) Change in distance between the COM of loop 465-474 and COM of Arg87 in GTP_cPEPCK and OAA complex simulation trajectory. H) Change in distance between the COM of loop 465-474 and COM of OAA in GTP_cPEPCK and OAA complex simulation trajectory.
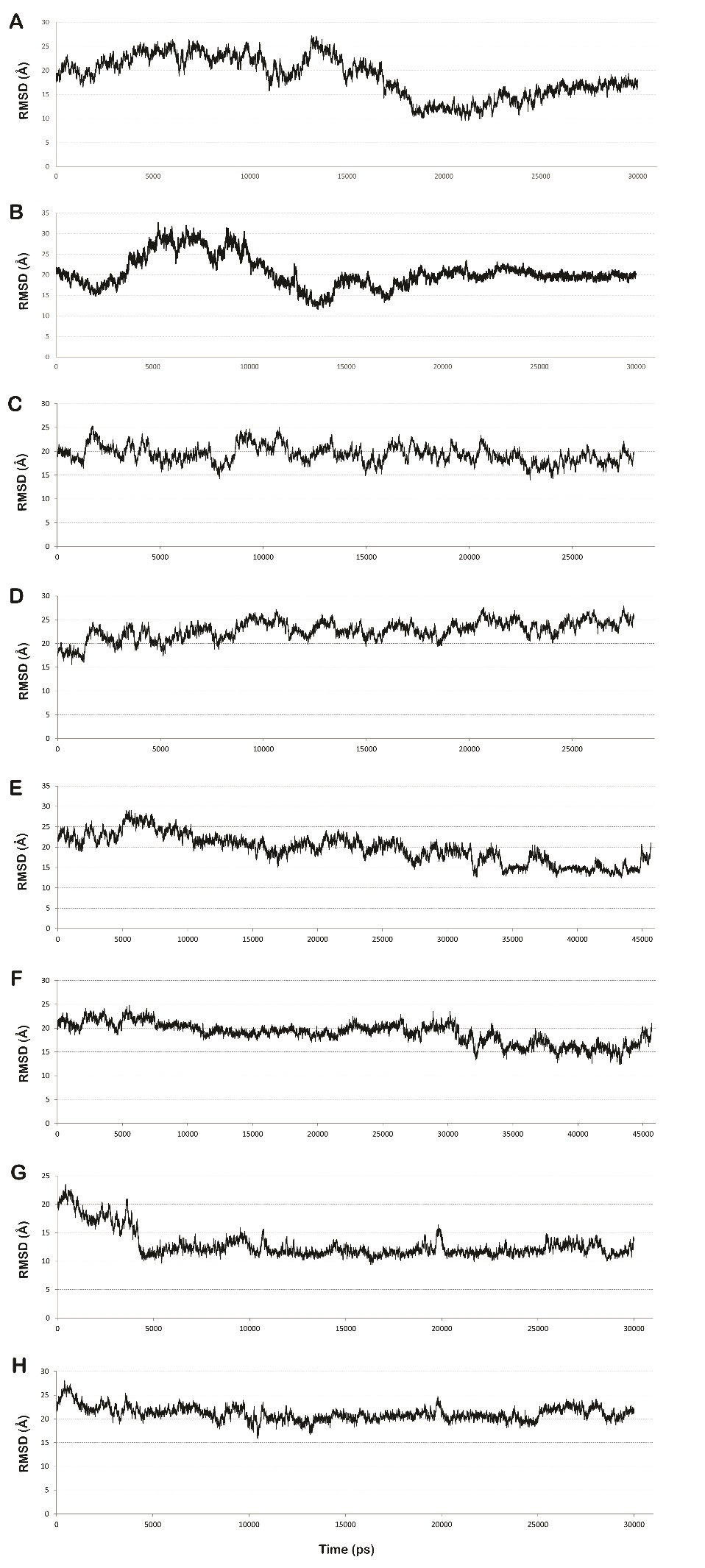
The distance analysis between COM of loop 465-474, Arg87, and genistein/OAA allowed us to correlate the movement of substrate/inhibitor at the binding site and movement of loop 465-474. In u_cPEPCK, the distance between COM of genistein and COM of loop 465-474 firstly increased and then returned back to its initial distance, which was 20Å. On the other hand, the distance between COM of Arg87 and COM of loop 465-474 decreased during the simulation by ~5Å. These results indicate that in u_cPEPCK, genistein shifted at GTP binding site which was at equal distance from the loop and its initial position. In GTP_cPEPCK, the distance between COM of Arg87 and COM of loop 465-474 remained constant but the distance between genistein and loop 465-474 increased by ~4-5 Å, which is justified as genistein moved towards extended binding site. In GDP_cPEPCK, distance between COM of Arg87 and COM of loop 465-474 as well as COM of genistein and COM of loop 465-474 were similar to u_cPEPCK. In GTP_cPEPCK and OAA complex, loop 465-474 came closer to Arg87 as well as to OAA. Hence, the closest association between Arg87 and loop 465-474 was observed when OAA was bound with cPEPCK.

**
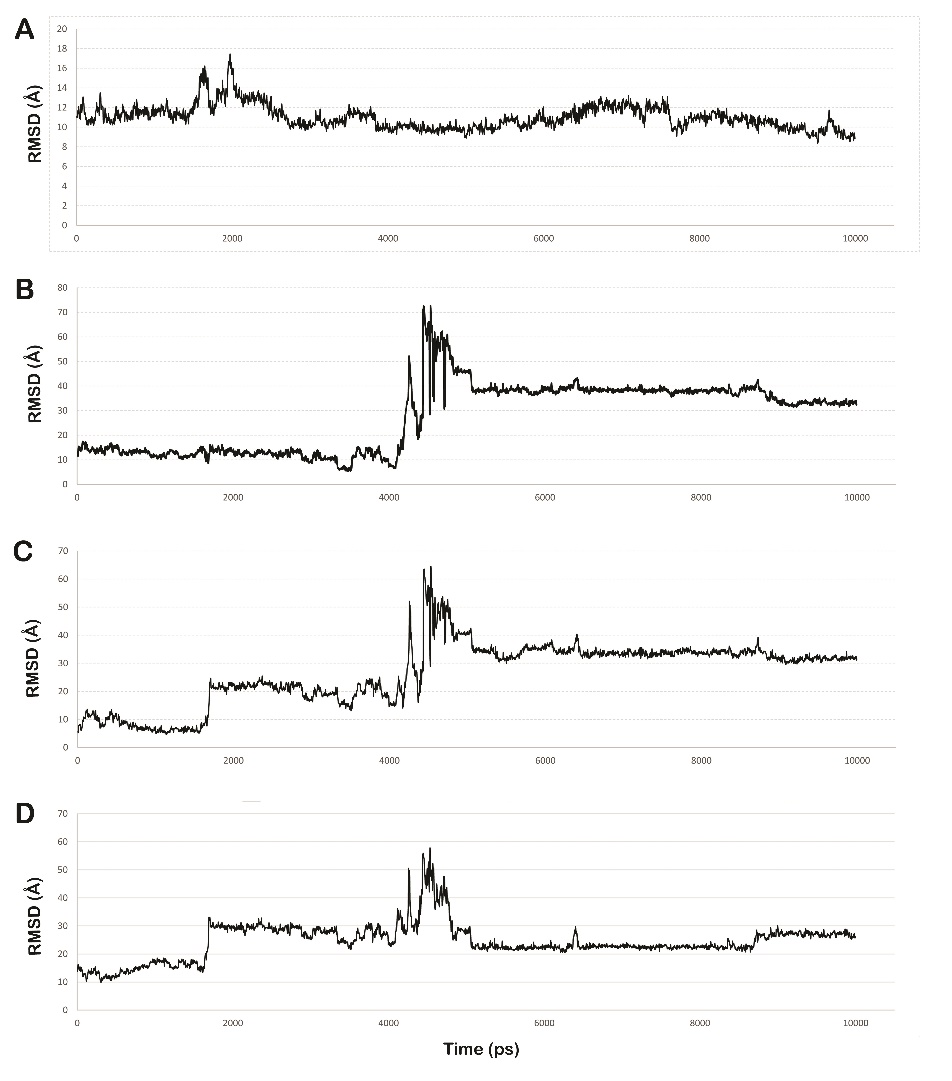
 S1 Appendix-Figure 7: Fluctuation in distance (Å) between COM of OAA and cPEPCK substrate binding site residues during simulation of GTP_OAA_cPEPCK and genistein complex.** A) Change in distance between the COM of loop 465-474 and COM of Arg87. B) Change in distance between the COM of loop 465-474 and COM of OAA. C) Change in distance between the COM of OAA and COM of Arg87. D) Change in distance between the COM of OAA and COM of Arg249.

The distance between COM of OAA and Arg87, Arg249 and loop 465-474 increased drastically after ~1.8 ns simulation, which indicates the release of OAA from the substrate binding site.

**
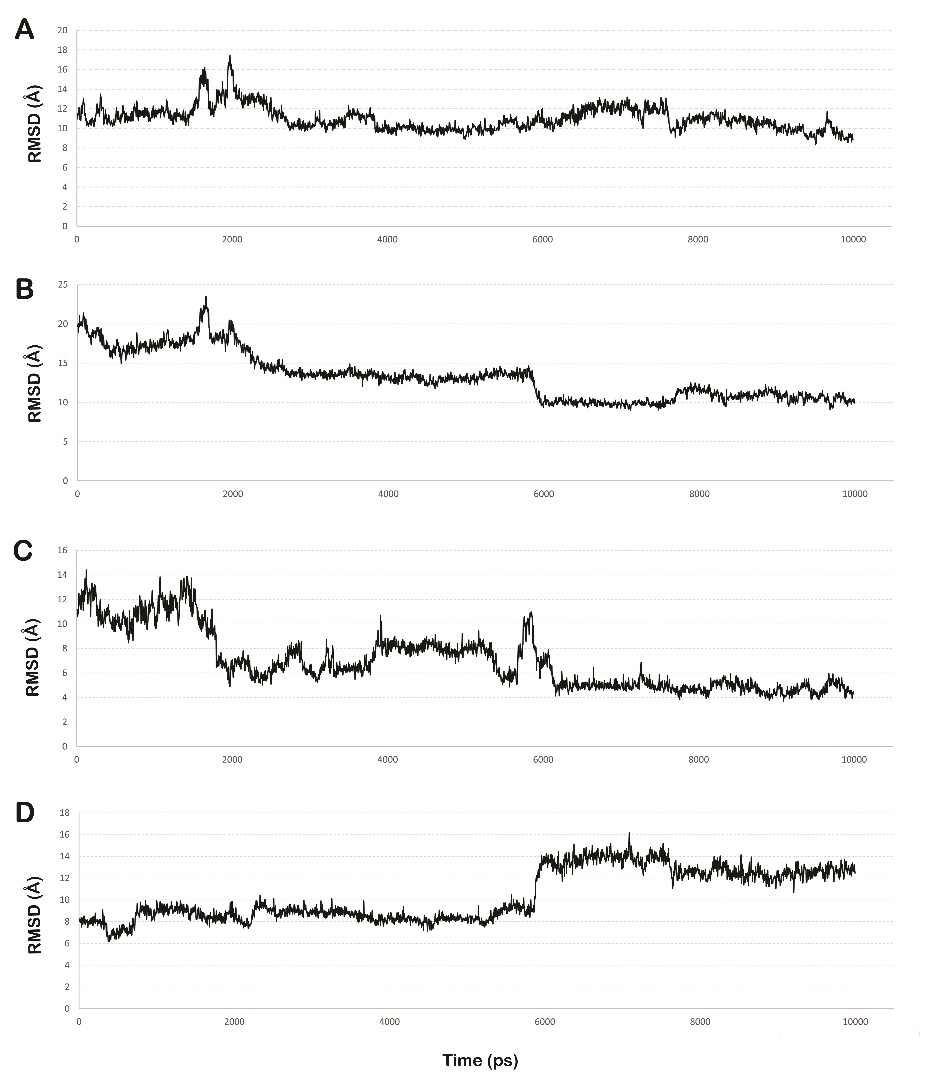
 S1 Appendix-Figure 8: Fluctuation in distance (Å) between COM of genistein and cPEPCK substrate binding site residues during simulation of GTP_OAA_cPEPCK and genistein complex.** A) Change in distance between the COM of loop 465-474 and COM of Arg87. B) Change in distance between the COM of loop 465-474 and COM of genistein. C) Change in distance between the COM of genistein and COM of Arg87. D) Change in distance between the COM of genistein and COM of Arg249.

The distance between COM of genistein and COM of Arg87 and loop 465-474 reduced and the distance between COM of genistein and COM of Arg249 increased, indicating the shift of genistein out of extended binding site.
