# Supplementary figures and images for: Mixed Inhibition of cPEPCK by Genistein, Using an Extended Binding Site Located Adjacent to Its Catalytic Cleft
Source: PLoS One. 2015 Nov 3;10(11):e0141987. doi: 10.1371/journal.pone.0141987 (PMC4631375; doi:10.1371/journal.pone.0141987)

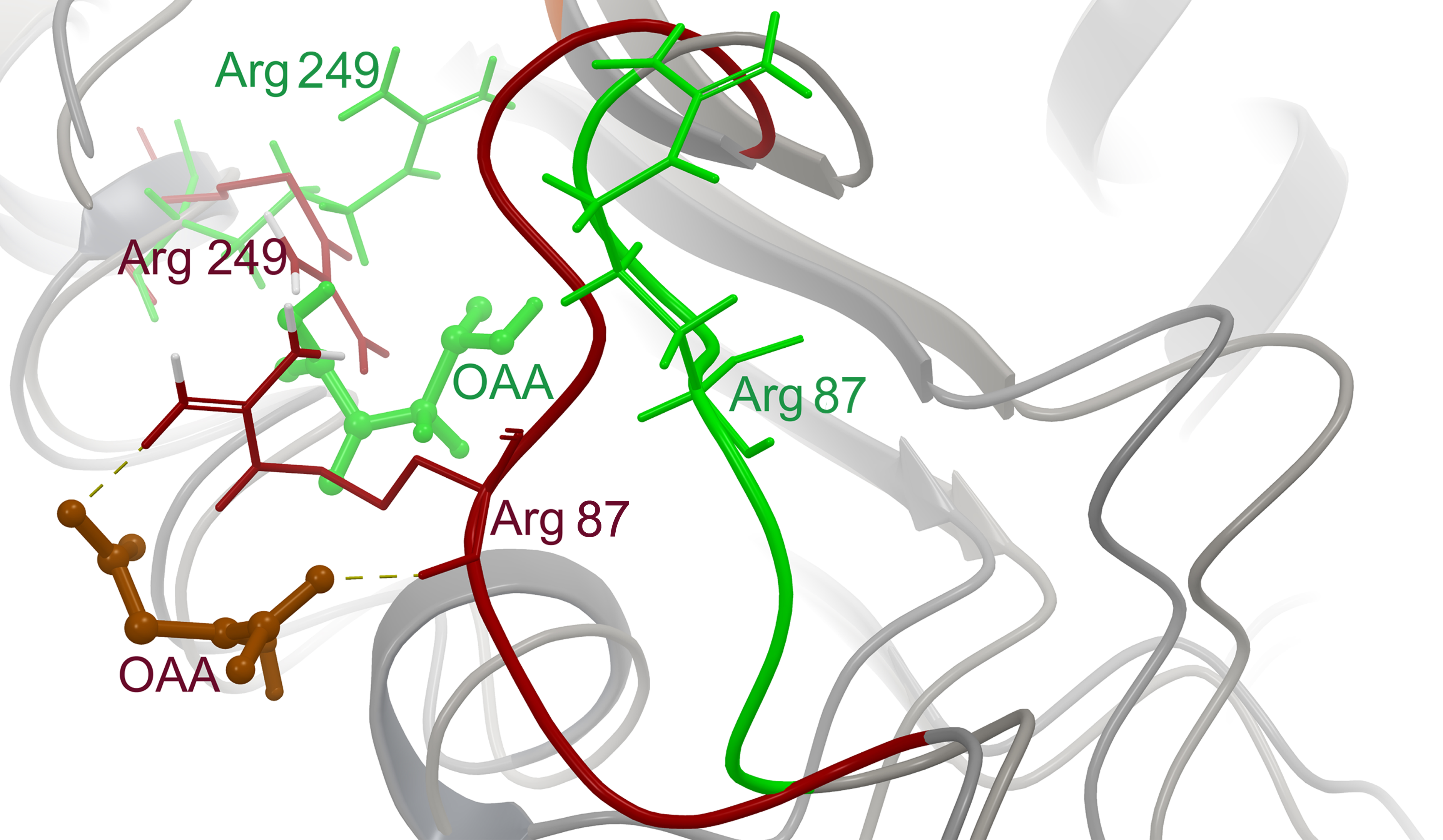

Supplement: S1 Fig — A) u_cPEPCK (green), GTP_cPEPCK (red) and GDP_cPEPCK (blue) are superimposed on each other to depict the changes in GTP binding site residues after energy minimization. B) 2D representation of interactions between GTP and its binding site residues in cPEPCK. (TIFF) [file pone.0141987.s002.tiff]

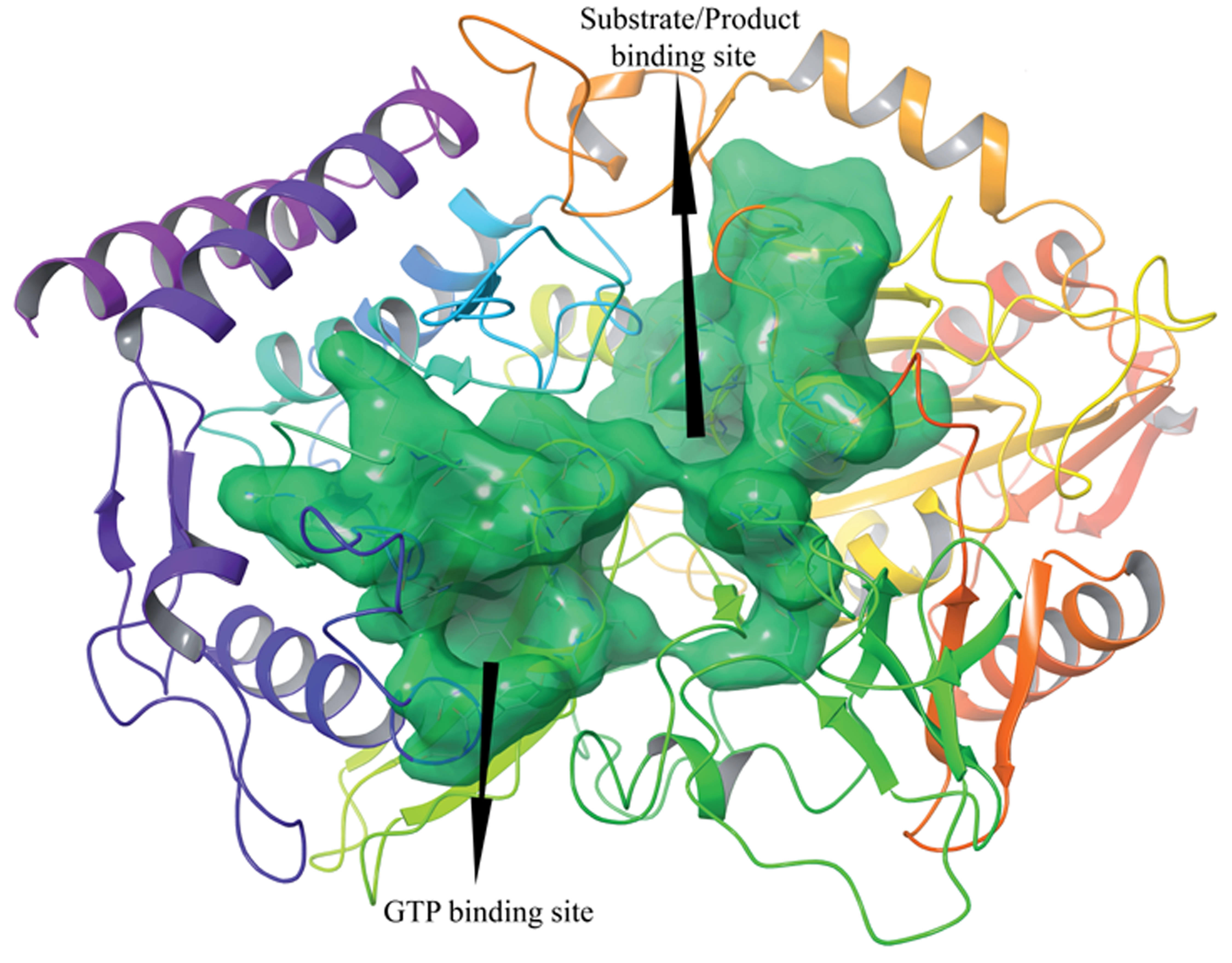

Supplement: S2 Fig — (TIFF) [file pone.0141987.s003.tiff]

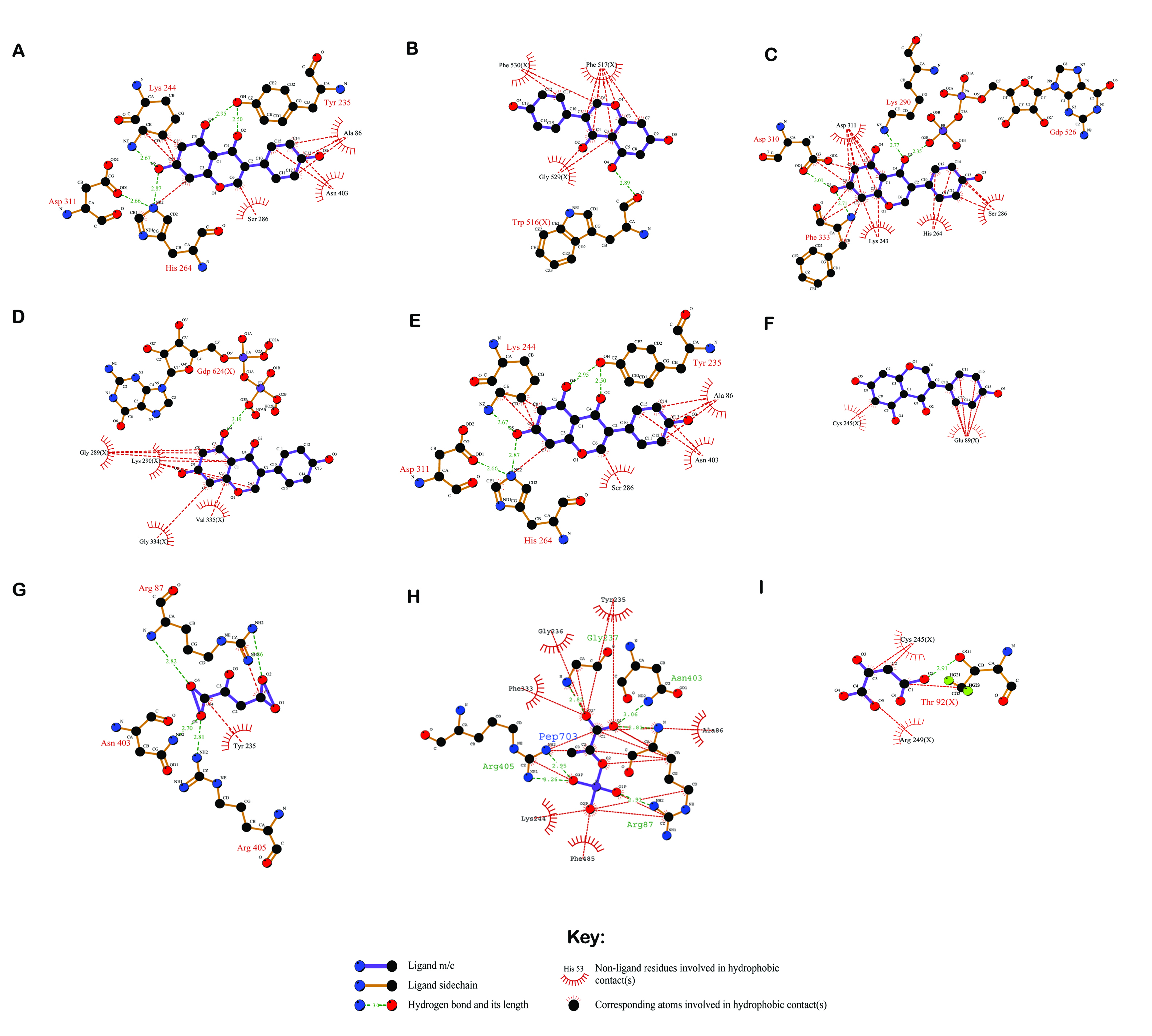

Supplement: S3 Fig — A) Interactions between u_cPEPCK and genistein before the 30 ns MD simulation. B) Interactions between u_cPEPCK and genistein after the 30 ns MD simulation. C) Interactions between GTP_cPEPCK and genistein before the 30 ns MD simulation. D) Interactions between GTP_cPEPCK and genistein after the 30 ns MD simulation. E) Interactions between GDP_cPEPCK and genistein before the 30 ns MD simulation. F) Interactions between GDP_cPEPCK and genistein after the 30 ns MD simulation. G) Interactions between GTP_cPEPCK and OAA before the 30 ns MD simulation. H) Interactions between GTP_cPEPCK and OAA after the 30 ns MD simulation. I) Interactions between GTP_cPEPCK and PEP after molecular docking. (TIFF) [file pone.0141987.s004.tiff]

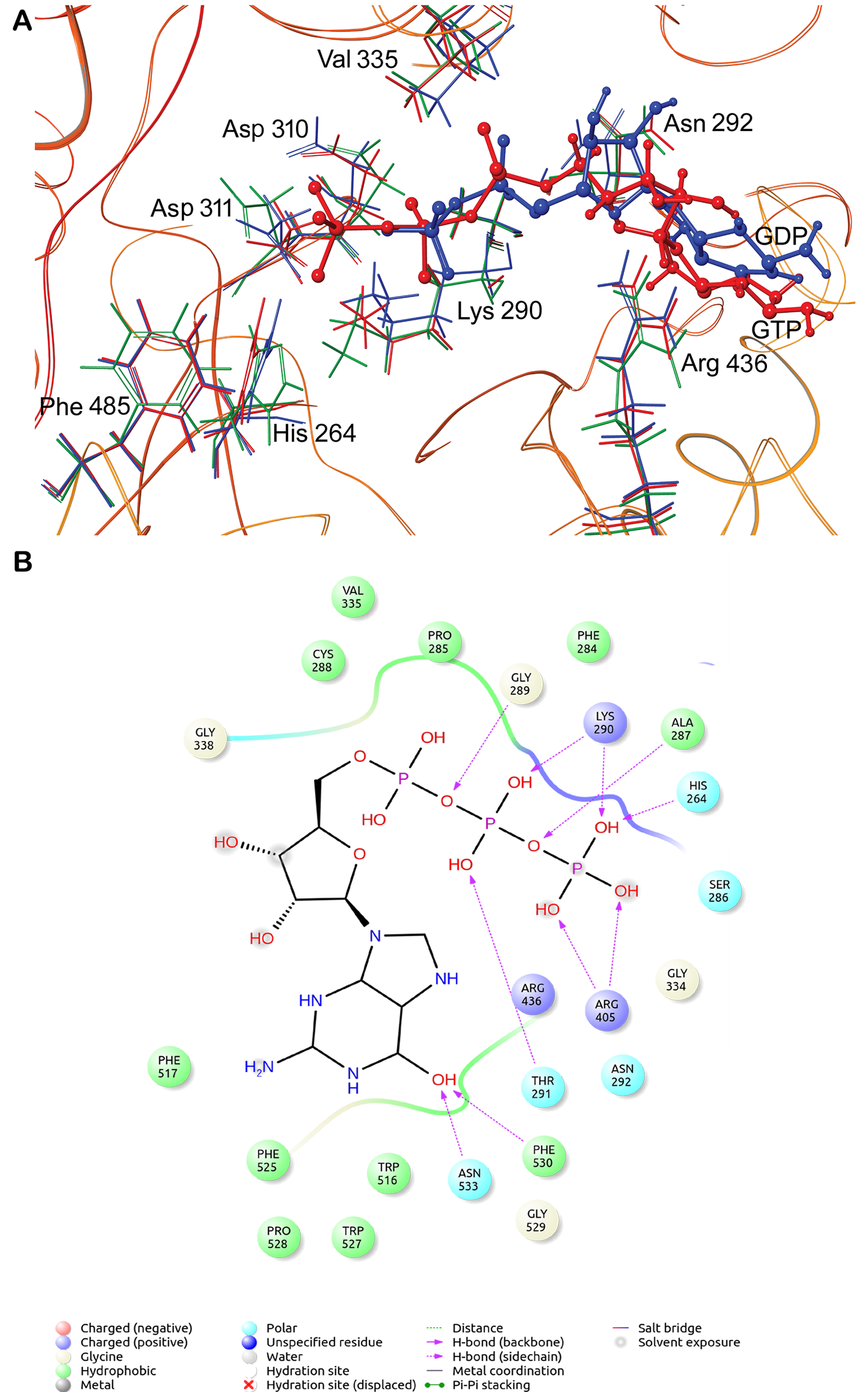

Supplement: S4 Fig — Initially when OAA is binding at substrate binding site, Arg87 has blocked the entrance of extended binding site while Arg249 has occupied the extended site (Red/Brown colored). During the simulation, Arg87 moved aside and Arg249 has also changed its conformations and OAA has shifted to the extended site (Green colored). (TIFF) [file pone.0141987.s005.tiff]

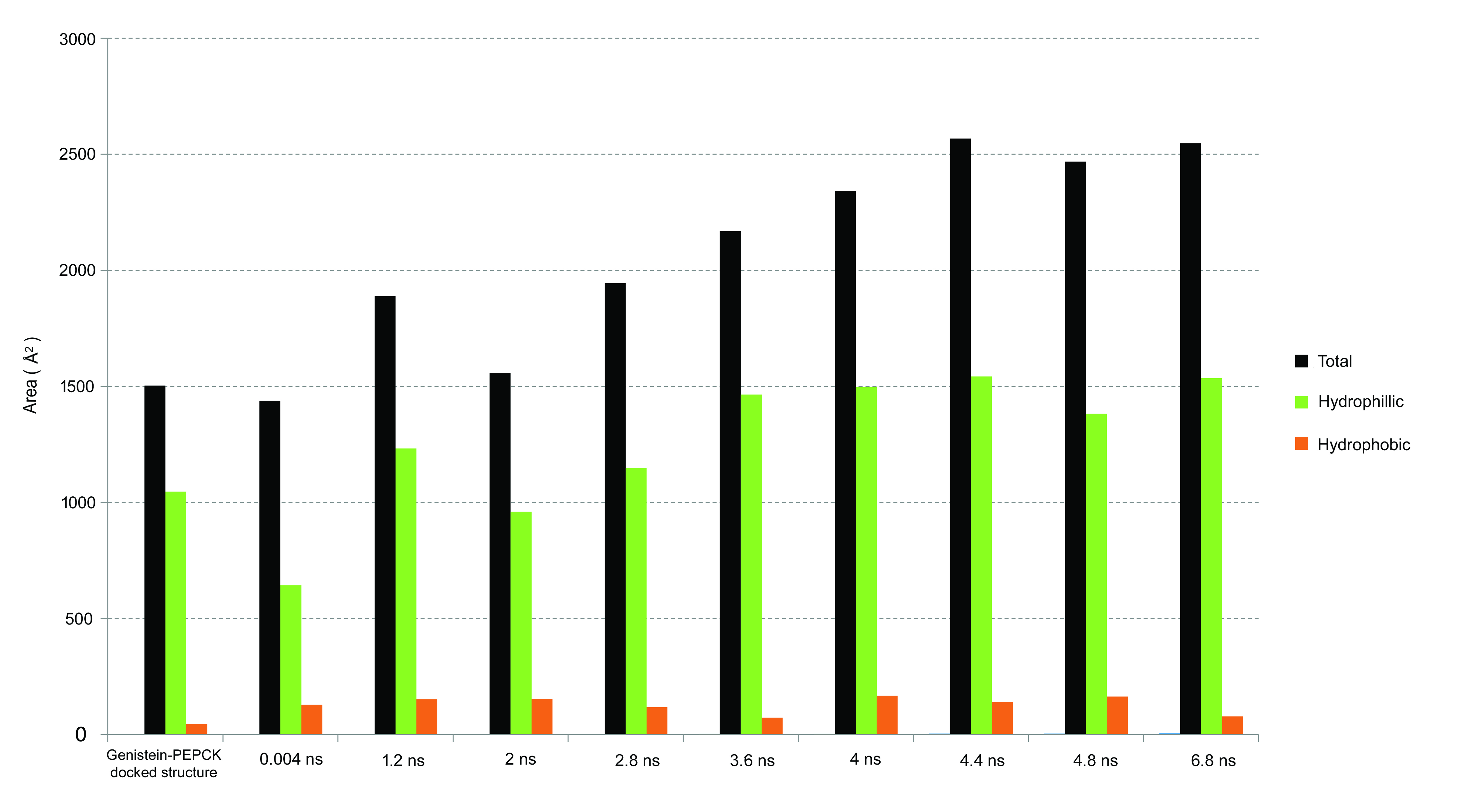

Supplement: S5 Fig — Black bars represent the total area, green represents the hydrophilic area and orange indicates the hydrophobic area. The first bar is for the area of extended site of the docked genistein GTP_cPEPCK complex. Genistein was docked at the substrate binding site and the area was calculated for the available binding space around 6Å of genistein. Later, as the simulation proceeded and genistein started moving into the extended binding site, its area also increased. Also, as the total area increased, hydrophilic area also increased. (TIFF) [file pone.0141987.s006.tiff]

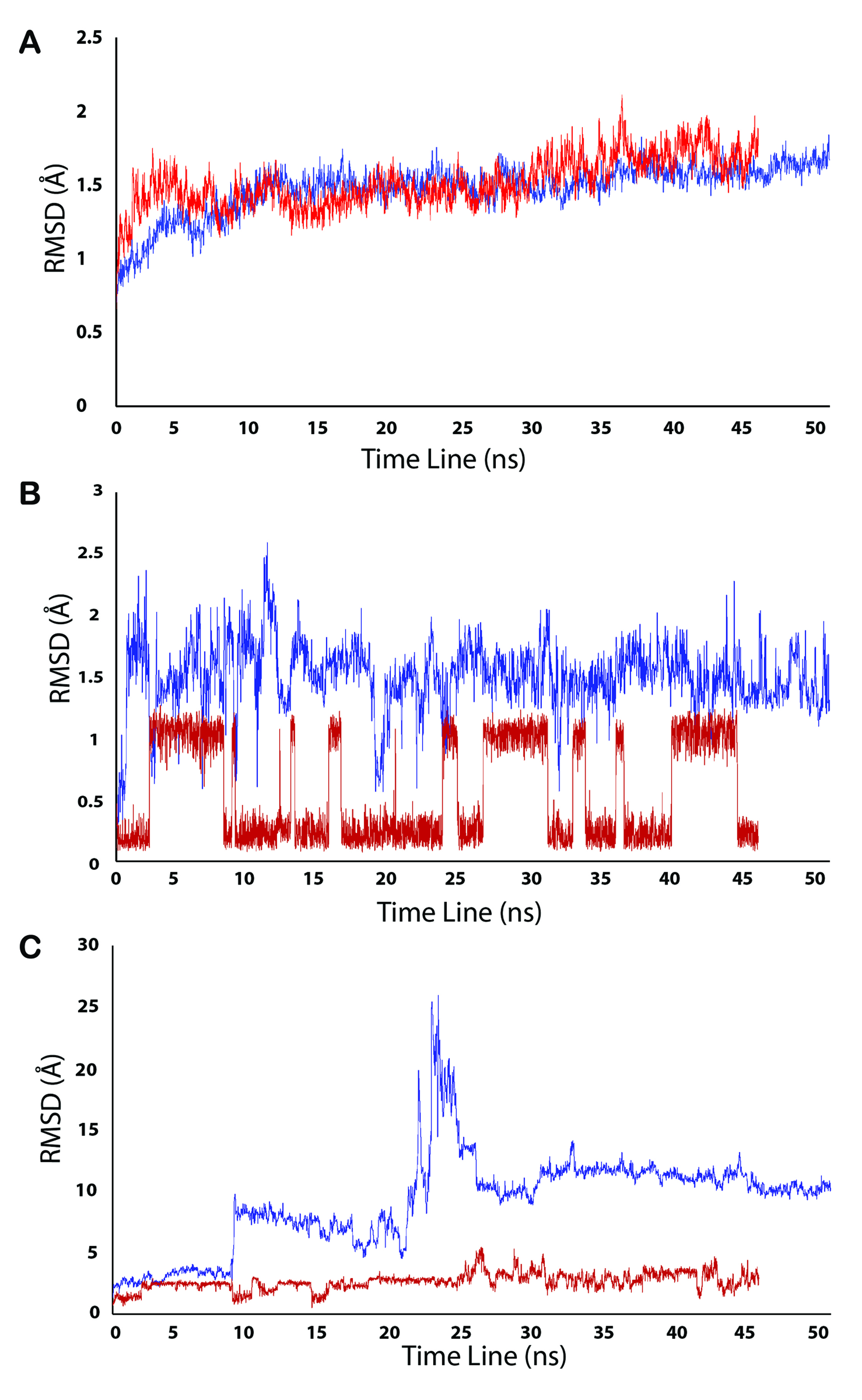

Supplement: S6 Fig — A) RMSD in cPEPCK backbone in the presence of 3-MPA (Red) and genistein (Blue) at extended sites. B) RMSD of 3-MPA (Red) and genistein (Blue) during their MD simulation at extended sites. Genistein did not move after it was stabilized at 2ns simulation time. 3-MPA is having less RMSD than genistein but it tends to change its conformation at intervals. C) Unstable OAA in the presence of 3-MPA or genistein at the extended site is displaying very high RMSD values. In the presence of 3-MPA (Red), OAA was moving within the binding site but could not leave the binding site while in the presence of Genistein (Blue), OAA left the binding site at 10ns simulation time. (TIFF) [file pone.0141987.s007.tiff]

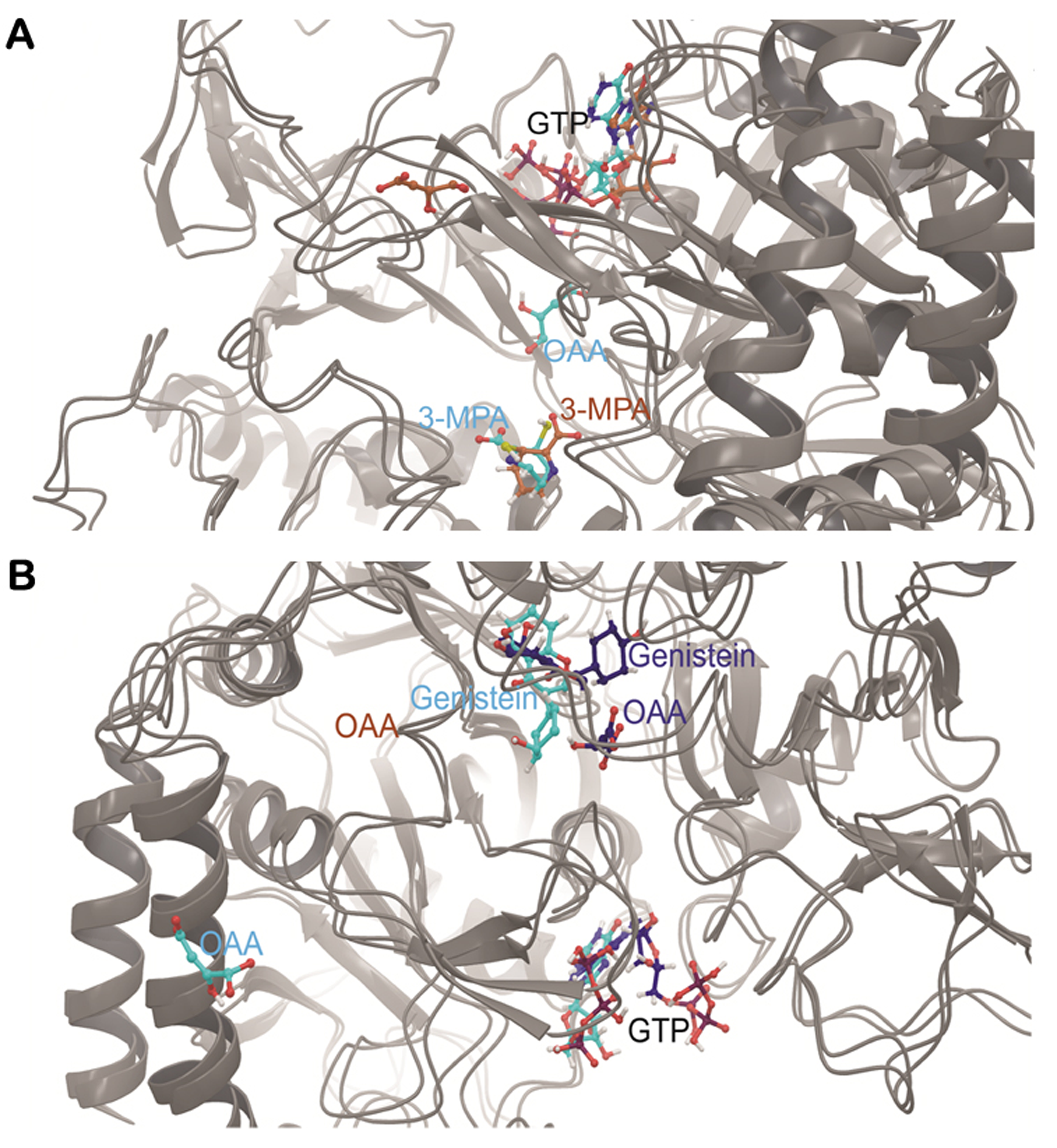

Supplement: S7 Fig — A) Initially OAA is present at its binding site and 3-MPA is bound adjacent to it (Cyan carbon skeleton). But during 45ns MD simulation, unstable OAA is trying to exit the cPEPCK binding site while 3-MPA still sits at the same extended pocket. B) Genistein is docked at the extended binding site, adjacent to OAA (Blue colored carbon skeleton). But during its 50ns MD simulation, unstable OAA actually has left the binding pocket of cPEPCK while genistein is still present at the extended site (Cyan colored carbon skeleton). Genistein’s phenol tail has changed its position and has shifted to OAA binding site. (TIFF) [file pone.0141987.s008.tiff]
